# Intelligent gold nanocluster for effective treatment of malignant tumor via tumor-specific photothermal–chemodynamic therapy with AIE guidance

**DOI:** 10.1093/nsr/nwae113

**Published:** 2024-03-22

**Authors:** Feng Liu, Tianfeng Yang, Xiaowei Chang, Li Chen, Cheng Cheng, Xiuhong Peng, Haihu Liu, Yanmin Zhang, Xin Chen

**Affiliations:** School of Energy and Power Engineering, Xi'an Jiaotong University, Xi'an 710049, China; Department of Chemical Engineering, Shaanxi Key Laboratory of Energy Chemical Process Intensification, Institute of Polymer Science in Chemical Engineering, School of Chemical Engineering and Technology, Xi'an Jiaotong University, Xi'an 710049, China; School of Pharmacy, Health Science Center, Xi'an Jiaotong University, Xi'an 710061, China; Department of Chemical Engineering, Shaanxi Key Laboratory of Energy Chemical Process Intensification, Institute of Polymer Science in Chemical Engineering, School of Chemical Engineering and Technology, Xi'an Jiaotong University, Xi'an 710049, China; Department of Chemical Engineering, Shaanxi Key Laboratory of Energy Chemical Process Intensification, Institute of Polymer Science in Chemical Engineering, School of Chemical Engineering and Technology, Xi'an Jiaotong University, Xi'an 710049, China; School of Pharmacy, Health Science Center, Xi'an Jiaotong University, Xi'an 710061, China; School of Pharmacy, Health Science Center, Xi'an Jiaotong University, Xi'an 710061, China; School of Energy and Power Engineering, Xi'an Jiaotong University, Xi'an 710049, China; School of Pharmacy, Health Science Center, Xi'an Jiaotong University, Xi'an 710061, China; Department of Chemical Engineering, Shaanxi Key Laboratory of Energy Chemical Process Intensification, Institute of Polymer Science in Chemical Engineering, School of Chemical Engineering and Technology, Xi'an Jiaotong University, Xi'an 710049, China

**Keywords:** AIE-guided precise tumor treatment, copper-dependent photothermal therapy, copper-triggered chemodynamic therapy, primary tumor elimination, copper-plunder-induced metastasis inhibition

## Abstract

Precise and efficient therapy of malignant tumors is always a challenge. Herein, gold nanoclusters co-modified by aggregation-induced-emission (AIE) molecules, copper ion chelator (acylthiourea) and tumor-targeting agent (folic acid) were fabricated to perform AIE-guided and tumor-specific synergistic therapy with great spatio-temporal controllability for the targeted elimination and metastasis inhibition of malignant tumors. During therapy, the functional gold nanoclusters (AuNTF) would rapidly accumulate in the tumor tissue due to the enhanced permeability and retention effect as well as folic acid-mediated tumor targeting, which was followed by endocytosis by tumor cells. After that, the overexpressed copper ions in the tumor cells would trigger the aggregation of these intracellular AuNTF via a chelation process that not only generated the photothermal agent *in situ* to perform the tumor-specific photothermal therapy damaging the primary tumor, but also led to the copper deficiency of tumor cells to inhibit its metastasis. Moreover, the copper ions were reduced to cuprous ions along with the chelation, which further catalysed the excess H_2_O_2_ in the tumor cells to produce cytotoxic reactive oxygen species, resulting in additional chemodynamic therapy for enhanced antitumor efficiency. The aggregation of AuNTF also activated the AIE molecules to present fluorescence, which not only imaged the therapeutic area for real-time monitoring of this tumor-specific synergistic therapy, but also allowed us to perform near-infrared radiation at the correct time point and location to achieve optimal photothermal therapy. Both *in vitro* and *in vivo* results revealed the strong tumor elimination, effective metastasis inhibition and high survival rate of tumor-bearing mice after treatment using the AuNTF nanoclusters, indicating that this AIE-guided and tumor-specific synergistic strategy could offer a promising approach for tumor therapy.

## Teaser

This work reports an intelligent gold nanocluster to perform precise photothermal-chemodynamic therapy with AIE guidance and on-demand manipulation of intracellular environments for integrative inhibition of primary tumor and its metastasis.

## INTRODUCTION

According to the statistics from the World Health Organization, malignant tumors have become one of the most serious diseases threatening human health [1]. Although conventional strategies such as surgical operation, radiotherapy and chemotherapy are widely used for the treatment of malignant tumors, the resulting tissue injury as well as the contradiction between therapeutic efficiency and systemic toxicity have all limited their application [2–5].

As to address above issues, some novel therapeutic strategies with good controllability based on nontoxic nanomaterials including photodynamic therapy, chemodynamic therapy, photothermal therapy and their combinative therapy were developed to achieve safe and effective treatment of malignant tumors [6–10]. Among these strategies, photothermal therapy relying on bio-safe photothermal agents have attracted plenty of attention due to its broad antitumor effect and easy operation, which could only be activated under near-infrared (NIR) irradiation, resulting in effective tumor therapy with barely any systemic toxicity [11–15]. However, considering the large biodistribution of photothermal agents around tumor tissue even after modification by tumor-targeting molecules [16–18], the constant photothermal conversion of conventional photothermal agents, regardless of their location, may cause serious damage to surrounding healthy tissue and immune cells in/around tumor tissue, thus leading to various unexpected side effects [19–21], especially when the tumors occur in important organs. Therefore, our lab and other research groups across the world have developed a series of stimuli-responsive gold nanoparticles. These gold nanoparticles can utilize endogenous conditions in the tumor to achieve aggregation only in the tumor tissue/cells where their photothermal properties would be activated for tumor-specific photothermal therapy [22–25], hopefully enhancing the therapeutic efficiency and reducing unexpected side effects for safe treatment.

Although this tumor microenvironment that is dependent on gold nanoparticles has opened a window for precise photothermal therapy, it is mainly focused on direct damage to the primary tumor, overlooking migration inhibition—one of the most important factors of tumor metastasis and recurrence [26–28]. Moreover, due to the invisible therapeutic process, it is a great challenge to perform the infrared irradiation at the correct time point and location to achieve optimal photothermal therapy, resulting in unstable treatments with relatively low efficiency, which limit its application in clinics. Although some fluorescent dyes have been used to label conventional photothermal agents in order to allow the visualization of therapy [29,30], these normal dyes can only display the location instead of the structural state of the photothermal agents and are invalid for recognizing aggregated gold nanoparticles with active photothermal properties. Therefore, to promote the application of photothermal therapy, it is required to develop novel intelligent gold nanoparticles that not only simultaneously achieve tumor microenvironment-dependent photothermal properties and effective inhibition of metastasis for tumor treatment, but also perform real-time imaging in accordance with the structure and function of these gold nanoparticles to precisely guide the therapeutic process.

Aggregation-induced-emission (AIE) fluorescent molecules have strong fluorescence in, and only in, the aggregate state due to the limitation of intramolecular motion [31–33]. They are able to exhibit an excellent linear concentration-dependent increase in brightness, strong resistance to photobleaching and low *in vivo* toxicity [34,35], and are therefore considered very promising candidates as fluorescence imaging agents for guiding various tumor therapies [36,37]. Moreover, their aggregation-dependent fluorescence exactly fits the requirements for the real-time imaging of the structure and function of gold nanoparticles for accurate photothermal therapy.

Recently, it has been reported that the abnormally high level of copper ions in tumor cells plays a critical role in metastasis, stimulating various metastasis-relative progressions such as proliferation, migration and vascularization [38,39]. Therefore, we believe that gold nanoparticles will be able to plunder the overexpressed copper ions in tumor cells while performing copper-induced aggregation and would be an ideal candidate for tumor-specific photothermal therapy and the effective inhibition of tumor metastasis. Moreover, a clear display of the accumulation and activation of these gold nanoparticles in tumor cells by using an aggregation-dependent imaging technique would further enhance the accuracy and efficiency of the treatment.

In addition, copper has been widely used as a catalytic agent to generate highly toxic hydroxyl radicals (•OH) via Fenton reactions with the overexpressed H_2_O_2_ in tumor tissue for antitumor chemodynamic therapy (CDT) [40]; it is able to exhibit extremely high Fenton catalytic activity and excellent CDT efficacy due to its large metal atom utilization efficiency and clear electronic structure [41]. Therefore, the effective utilization and manipulation of copper in tumor cells by using engineered gold nanoparticles would not only induce the copper-mediated photothermal therapy to specifically damage the primary tumor and suppress its metastasis, but also perform additional chemodynamic therapy to further enhance the therapeutic efficacy.

**Figure 1.**
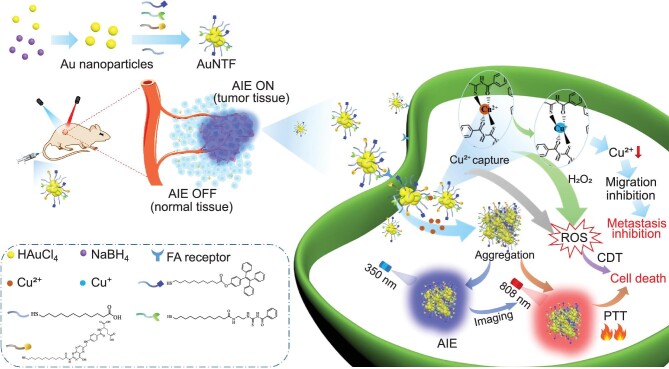
Schematic representation of the synthesis and therapeutic mechanism of AuNTF nanoclusters. The nanoclusters are able to utilize and manipulate the overexpressed copper ions in tumor cells for the simultaneous inhibition of the primary tumor and its metastasis via accurate and visible synergistic therapy with AIE guidance.

Inspired by the above ideas, we developed acylthiourea (copper ions chelator), fluorescent molecule with AIE performance (imaging agent) and folic acid (tumor-targeting agent) co-modified gold nanoclusters (AuNTF) to perform tumor-specific synergistic therapy with the real-time guidance of AIE imaging for the effective treatment of a malignant tumor. The folic acid was expected to medicate the accumulation of the AuNTF nanoclusters in the tumor cells [42], while the acylthiourea was designed to capture the overexpressed copper ions and connect the AuNTF nanoclusters via ion chelation [39,43]. This would generate the photothermal agent in tumor cells to damage the tumor under NIR radiation and simultaneously perform copper deficiency to inhibit tumor metastasis. Because of the high reducibility of acylthiourea, the captured copper ions were further reduced to cuprous ions, which served as a catalyst to convert the excess H_2_O_2_ into toxic reactive oxygen species (ROS) in tumor cells [44,45], resulting in additional chemodynamic therapy to promote the therapeutic efficiency. Moreover, due to the existence of AIE molecules, the copper-induced aggregation of the AuNTF nanoclusters would be clearly monitored by using fluorescence imaging [46–48], which is the starting point for photothermal therapy, chemodynamic therapy and copper deficiency, allowing us to visualize all these therapeutic processes while performing optimal photothermal therapy via the correct introduction of NIR radiation on time (Fig. 1).

## RESULTS AND DISCUSSION

### Fabrication and characterization of AuNTF nanoclusters

**Figure 2.**
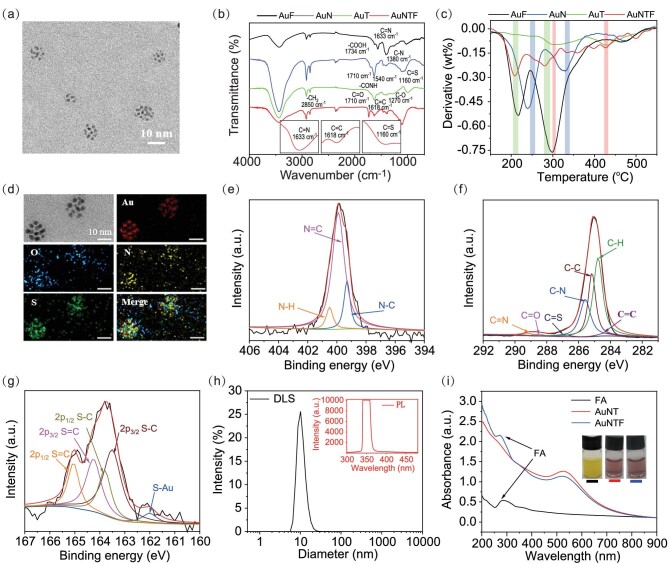
(a) Transmission electron microscopy (TEM) images of AuNTF nanoclusters. (b) Infrared (IR) spectra and (c) derivative thermogravimetry (DTG) analysis of FA-modified gold nanoclusters (AuF), NACB-modified gold nanoclusters (AuN), TPE-modified gold nanoclusters (AuT) and AuNTF nanoclusters. (d) Energy dispersive spectroscopy (EDS) mapping images of AuNTF nanoclusters. (e–g) X-ray photoelectron spectroscopy (XPS) of (e) N 1s, (f) C 1s and (g) S 2p obtained from AuNTF nanoclusters. (h) Dynamic light scattering (DLS, black curve) and fluorescence spectra (red curve) of AuNTF nanoclusters, where the excitation wavelength was 350 nm. (i) UV–Vis spectra and photographs of FA solution, NACB and TPE co-modified gold nanoclusters (AuNT) solution and AuNTF solution.

To prepare the AuNTF nanoclusters, thiol functionalized 4-(1,2,2-triphenylethenyl)phenol (TPE-SH, AIE agent), thiol functionalized *N-*((2-aminoethy)carbamothioyl) benzamide (NACB-SH, copper ions chelator) and thiol functionalized folic acid (FA-SH, targeting agent) were synthesized and characterized by using ^1^H NMR (Fig. S1). As expected, strong fluorescence appeared once the TPE-SH switched from monodisperse state to aggregated state, indicating its great AIE performance (Fig. S2). The resulting TPE-SH, NACB-SH and FA-SH were further modified on the surface of pre-synthesized gold nanoparticles at a mole ratio of 2:4:1 through Au–S bonds to produce the AuNTF nanoclusters. As can be seen from Fig. 2a, the AuNTF nanoclusters consisted of several nanoparticles, which distributed well in aqueous solution with an average size of ∼10 nm.

The synthesis process of AuNTF was first investigated by using infrared (IR) spectra (Fig. 2b) and derivative thermogravimetry (DTG) analysis (Fig. 2c), which showed the characteristic vibrations (C=S from NACB, C=C from TPE and C=N from FA) as well as all thermogravimetric peaks of TPE, NACB and FA in the group of AuNTF nanoclusters, indicating the successful introduction of the designed functional molecules. The chemical components of the resulting AuNTF nanoclusters were further investigated by using energy dispersive X-ray spectroscopy (EDS, Fig. 2d). As can be seen from this figure, characteristic elements of AuNPs, TPE, NACB and FA including Au, S, N and O were all observed as expected. In addition, all the organic elements completely co-located with Au, indicating the uniform modification of AuNTF. Further information about the modification layer was provided by using x-ray photoelectron spectroscopy (XPS, Fig. 2e–g), which showed the peaks of N 1s (C=N) at 399.3 eV and C 1s (C=N) at 289.1 eV from FA, the peak of C 1s (C=C) at 284.8 eV from TPE, as well as the peaks of S 2p (C=S) at 164.3 eV and C 1s (C=S) at 286.7 eV from NACB. All these results indicated the successful formation of AuNTF nanoclusters as designed.

Although the AuNTF were formed by the slight assembly of several nanoparticles, the nanocluster structure did not influence the optical property of either the AIE molecules or the gold nanoparticles in the AuNTF, which still performed the negligible fluorescence and a major characteristic absorption band at 520 nm similarly to the property of monodisperse AIE molecules and gold nanoparticles (Fig. 2h and i). The undetectable fluorescence and weak long-wavelength absorption of the AuNTF were expected to be significantly enhanced after the copper-induced aggregation, which would not only achieve the selective photothermal conversion under 808 nm radiation to avoid the unexpected damage of healthy tissue during photothermal therapy, but also allow the therapy to be visible for precise manipulation.

### Copper-dependent activation of AuNTF nanoclusters for tumor theranostics

**Figure 3.**
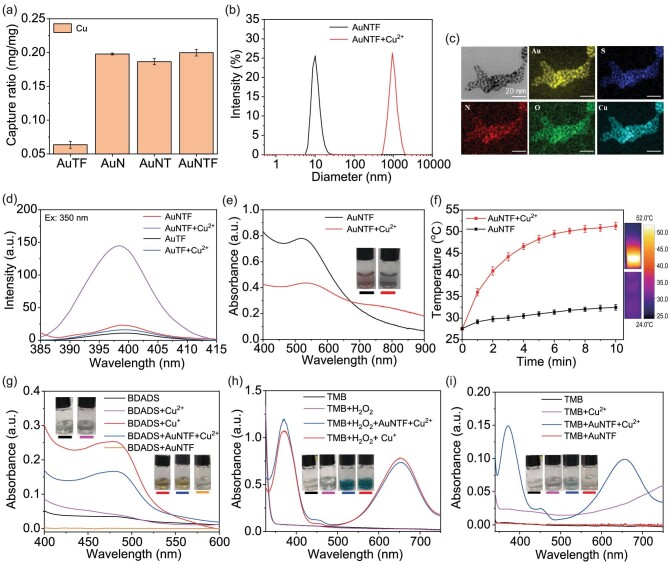
(a) Copper-capture properties of TPE- and FA-co-modified gold nanoclusters (AuTF), NACB-modified gold nanoclusters (AuN), NACB- and TPE-co-modified gold nanoclusters (AuNT) as well as AuNTF nanoclusters determined by using atomic absorption spectrometry. AuTF was used as a negative control. (b) Size difference of AuNTF nanoclusters in solution with and without Cu^2+^ measured by using DLS. (c) TEM and EDS mapping images of AuNTF nanoclusters after copper-induced aggregation. (d) Fluorescence spectra of AuNT and AuNTF in phosphate buffered saline (PBS) solution with and without Cu^2+^. (e) UV–Vis spectra and photographs of AuNTF in PBS solution with and without Cu^2+^. (f) Photothermal curves and photothermal images (10 min after irradiation) of the AuNTF in PBS solution with and without Cu^2+^; the radiation wavelength was 808 nm. (g) UV–Vis spectra and photographs of bathocuproine disulfonic acid disodium salt (BDADS, cuprous ion indicator) solution with and without Cu^2+^, Cu^+^, AuNTF as well as AuNTF + Cu^2+^ indicated the occurrence of copper reduction to generate cuprous ions during copper capture. (h) UV–Vis spectra and photographs of 3,3’,5,5’-tetramethylbenzidine (TMB, ROS indicator) solution with and without H_2_O_2_, H_2_O_2_ + Cu^+^ as well as H_2_O_2_ + AuNTF + Cu^2+^ indicated that the resulting cuprous ions would further catalyse H_2_O_2_ to produce ROS for chemodynamic therapy. (i) UV–Vis spectra and photographs of TMB solution with and without Cu^2+^, AuNTF as well as AuNTF + Cu^2+^ indicated that the ROS production also happened alongside the copper reduction.

According to the design, the photothermal performance, chemodynamic performance and fluorescence imaging performance of AuNTF nanoclusters all started from the copper capture. Therefore, the copper-capture ability of the AuNTF was investigated by using atomic absorption spectrometry. As shown in Fig. 3a, 1 mg of AuNTF could combine ≤0.2 mg of copper ions whether with or without the modification of FA and TPE, demonstrating the efficient copper pillage of the AuNTF from the surrounding environment due to the strong chelation between the NACB and copper ions, which could be further used to reduce the overexpressed copper ions in tumor cells for effective inhibition of copper-mediated metastasis.

As expected, the chelation further switched the structure of the AuNTF from monodisperse state to aggregation state (Fig. 3b), generating a large aggregate densely stacked by AuNTF in which the copper ions distributed well in the aggregate serving as cross-linking points due to the multiple coordination between the NACB and copper ions (Fig. 3c). The aggregation process not only closely packed AIE molecules to generate strong fluoresce at 400 nm for monitoring the copper-induced structure and function transformation of AuNTF (Fig. 3d), but also influenced the localized surface plasmon resonance of the gold nanoparticles, resulting in significant enhancement of the absorption in the long-wavelength region to perform the copper-dependent photothermal properties under 808 nm radiation (Fig. 3e and f). The negligible adsorption at 808 nm and weak photothermal effect of the pure copper ion chelators (NACB) in the with/without Cu^2+^ and AuTF + Cu^2+^ groups indicated that the enhancement of the photothermal effect caused by the copper ions themselves can be excluded (Fig. S3), with the effective and selective photothermal therapy attributed to the copper-triggered aggregation of the gold nanoparticles. In addition, the strong chelation between the copper ions and the AuNTF allowed repeatable photothermal cycles (Fig. S4) that are beneficial for the clinical applications of AuNTF, which normally require sustained therapy.

Besides the copper-induced optical performance change of the AuNTF, the activation of its chemodynamic properties after aggregation was also measured. As can be seen from Fig. 3g, the AuNTF was able to rapidly reduce the copper ions to cuprous ions along with the chelation, which could be detected by using the typical cuprous ions indicator (bathocuproine disulfonic acid disodium salt, BDADS)[49] and UV–Vis spectra. The cuprous ions generated *in situ* further served as a catalyst to convert the H_2_O_2_ into ROS, which was monitored by the color change of the ROS indicator [50,51] (Fig. 3h), hopefully resulting in highly selective chemodynamic therapy in tumor cells containing both excess copper ions and overexpressed H_2_O_2_. Moreover, the reduction process not only generated cuprous ions, but also produced ROS alongside the formation of cuprous ions (Fig. 3i), which led to an additional chemodynamic therapy to further enhance the therapeutic efficiency.

### Copper specificity and physiological stability of AuNTF nanoclusters

As mentioned above, the AuNTF nanoclusters were designed to perform a series of copper-dependent activities in tumor cells for tumor-specific therapy. Considering the possible interference of various ions in the physiological environment, the copper-capture property, photothermal property, chemodynamic property and fluorescence imaging of AuNTF nanoclusters in solution containing K^+^, Na^+^, Ca^2+^, Mg^2+^, Fe^2+^ and/or Cu^2+^ (major ions in the biosystem) were investigated. As can be seen from Fig. S5a, the AuNTF nanoclusters preferred to capture Cu^2+^ by far over other ions, followed by the effective generation of cuprous ions to perform chemodynamic therapy even under conditions with H_2_O_2_ and mixed ions (Fig. S5b). Due to the high selectivity of the AuNTF nanoclusters to copper ions, the aggregation also only relied on the existence of copper ions regardless of other ions (Fig. S5c). This process simultaneously led to the activation of AIE performance for fluorescence imaging (Fig. S5d) and an obvious rise in absorption in the long-wavelength region (Fig. S6) as long as the copper ions were added in the system, resulting in the expected copper-dependent photothermal conversion of AuNTF nanoclusters even under complex physiological conditions (Fig. S5e).

We further tested whether the endogenous concentration of Cu^2+^ in tumor cells could trigger AuNTF aggregation. The levels of copper ions in the tumor cells were tested and are shown in Fig. S7a. As can be seen from this figure, the concentration of copper ions in the tumor was ∼1 mg/L. In order to verify whether the 1 mg/L of Cu^2+^ was able to trigger the aggregation of our AuNTF nanoparticles, a transmission electron microscope (TEM) was used to monitor the 4T1 cells after 12 h of treatment using the AuNTF nanoparticles. As can be seen from Fig. S7b, significant aggregations of the nanoparticles were observed in tumor cells, which indicated that the endogenous Cu^2+^ in tumor cells is sufficient to aggregate the AuNTF nanoparticles for further therapy. More information was provided by the size distribution, absorption and photothermal properties of the AuNTF nanoparticles before and after incubation with 1 mg/L of Cu^2+^ (Fig. S7c–e), which performed significant enhancement of the particle size from 10 to >1000 nm with the addition of the Cu^2+^, resulting in a dramatic rise in the absorbance at 808 nm and >20 degrees of temperature promotion only after 10 min of 808 nm radiation. All these results indicated that the AuNTF nanoparticles could rapidly aggregate under the endogenous concentration of Cu^2+^ in tumor cells (1 mg/L) to perform effective photothermal therapy as designed.

Besides the copper-dependent photothermal therapy, the high stability of the AuNTF nanoclusters under physiological conditions is also important for maintaining their antitumor performance before arriving at the tumor tissue. As can be seen from Figs S8 and S9, the size distribution and absorption peak of the AuNTF nanoclusters was well maintained even after 48 h of incubation in phosphate buffered saline (PBS) (pH 7.4, simulated physiological environment), which showed the constant physical structure of AuNTF during long-term therapy. Moreover, the AuNTF nanoclusters after incubation still presented a high copper-capture ratio (Fig. S5f), effective copper reduction (Fig. S10) and a copper-reduction-medicated chemodynamic process (Figs S5g and S11) in which the 48 h of incubation in the PBS did not influence the efficiency. As expected, the copper capture further caused the aggregation of AuNTF nanoclusters even after 48 h of incubation (Fig. S8), which not only triggered the AIE performance of the AuNTF for fluorescence imaging to monitor the therapeutic processes (Fig. S5h), but also caused the significant red-shift of the absorption peak (Fig. S9), resulting in the copper-dependent photothermal conversion for tumor-specific photothermal therapy (Fig. S5i). All these results indicated that the AuNTF nanoclusters could maintain their structure and functions in a physiological environment for the long term to support the designed copper-dependent activities for tumor-specific theranostics.

### *In vitro* tumor-specific AIE property of AuNTF nanoclusters

Before measuring the antitumor performance of the AuNTF nanoclusters, their tumor-specific AIE property for real-time monitoring and proper guidance of the therapeutic process was investigated at the cellular level. In order to perform this experiment, FITC-labeled AuNTF was employed and co-incubated with human breast epithelial cells (MCF-10A, represented normal cells) and mouse breast cancer cells (4T1, represented tumor cells), followed by the evaluation using fluorescent imaging and flow cytometry analysis to demonstrate the tumor-specific uptake of the AuNTF nanoclusters at first. The nanoclusters without the modification of folic acid (AuNT) were used as a control group. The AuNTF and AuNT were labeled by equal amounts of FITC fluorescent molecules, which were 100 μg/mL for the intracellular experiments. The FITC labeling was used to detect the location of the AuNTF in or out of tumor/normal cells regardless of their aggregation state (Fig. S12), while the TPE labeling was used to detect the aggregation state of the AuNTF. It can also be seen from Fig. S13a and b that the tumor cells after incubation with AuNT presented much stronger fluorescence than that of normal cells due to the disparate copper concentration in these cells, where the overexpressed copper ions in the tumor cells triggered the aggregation of AuNT via chelation to enhance their retention, resulting in the tumor-selective accumulation of these nanoclusters. As expected, the fluorescence difference between the tumor cells and the normal cells was significantly magnified in the AuNTF group, indicating the further enhancement of the tumor selectivity after the introduction of FA, which could be attributed to the synergistic effect of the aggregation-promoted retention in the tumor cells and FA-mediated internalization by the tumor cells (overexpressed folate receptors in 4T1 cells). The quantitative analysis is presented in Fig. S13c and d. These results not only presented the prior uptake of AuNTF by tumor cells benefitting the tumor-specific therapy, but also provided evidence about the occurrence of copper-induced aggregation, which we believe would further activate the AIE performance to achieve fluorescence imaging for the monitoring and guidance of therapy.

After verifying the tumor-selective uptake of the AuNTF, the subsequent tumor-specific AIE performance of these nanoclusters was further investigated by using a fluorescent microscope. The PBS and nanoclusters without modification of FA (AuNT), TPE (AuNF) or NACB (AuTF) were used as control groups. As can be seen from Fig. S13e and f, no visible fluorescence was detected in the healthy cells (MCF-10A) after incubation with PBS and various nanoclusters. However, obvious fluorescence appeared in AuNT- and/or AuNTF-treated tumor cells (4T1), demonstrating the tumor-specific AIE performance of these two types of nanoclusters, which could be attributed to the close packing of the TPE (AIE agent) caused by the copper-induced nanoclusters aggregation. Moreover, the fluorescence in the AuNTF group is much stronger than that in the AuNT group due to the additional FA-mediated tumor targeting, which indicated the optimal candidate of AuNTF for tumor-specific AIE as designed. Therefore, the strong fluorescence of both FITC and AIE in tumor cells as well as the weak fluorescence of both FITC and AIE in normal cells not only indicated the specific uptake of AuNTF by tumor cells, but also showed that the aggregation of AuNTF only happened in tumor cells.

The real-time AIE performance of the AuNTF in tumor cells was also investigated by using a fluorescent microscope to present the dynamic aggregation of the AuNTF (Fig. S13g and h), which indicated that the aggregation of the AuNTF occurred in tumor cells after 6 h of incubation and the aggregation was almost completed in 10 h. Because the aggregation status of the AuNTF nanoclusters is the origin of all antitumor actions, the real-time AIE results could not only clearly monitor the therapeutic process, but also provide direct evidence to determine the time point of NIR radiation for optimal photothermal therapy.

### *In vitro* antitumor performance of AuNTF nanoclusters

**Figure 4.**
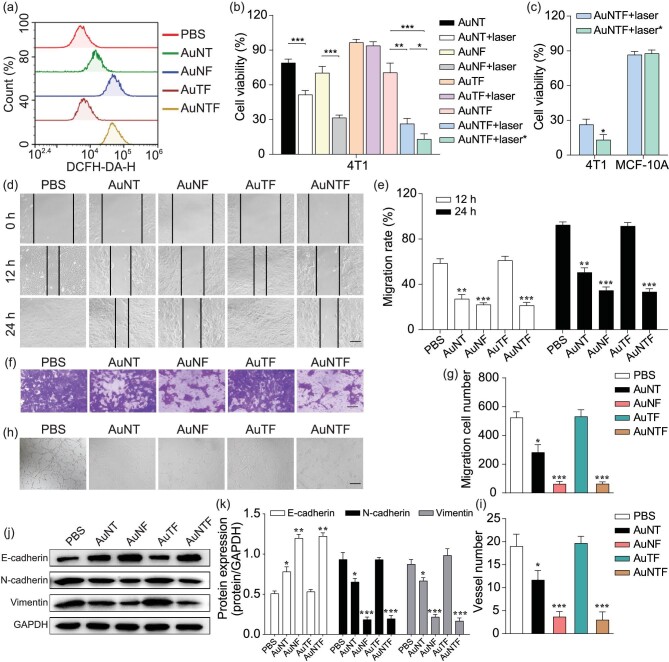
(a) ROS generation in 4T1 cells after 10 h of incubation with PBS, AuNT, AuNF, AuTF and AuNTF determined by using flow cytometry analysis with DCFH-DA dye staining. (b) Cell viability of 4T1 cells after treatment with various formulas. The laser indicated 808 nm NIR irradiation at 8 h after incubation (photothermal therapy without AIE guidance), while the laser* indicates 808 nm NIR irradiation at 10 h after incubation (photothermal therapy guided by AIE signal). (c) Comparison between cell viability of 4T1 cells and MCF-10A cells after treatment using AuNTF under NIR irradiation with and without AIE guidance. (d) Photographs of 4T1 cells to indicate their migration in scratch assay after treatments using various formulas. The scale bar is 200 μm. (e) Quantitative analysis of (d). (f) Images of 4T1 cells that migrated through the polycarbonate membrane, which were treated using various formulas and stained using 0.2% crystal violet. The scale bar is 100 µm. (g) Quantitative analysis of (f). (h) Tube-forming model images of HUVECs after treatment using various formulas. The scale bar is 100 µm. (i) Quantitative analysis of (h). (j) Protein expression of E-cadherin, N-cadherin and Vimentin in 4T1 cells after treatment using various formulas. (k) Quantitative analysis of (j). Data are presented as means ± SD (*n* = 3). The asterisks indicate that the differences between the control groups are statistically significant using an ANOVA test (*P < 0.05, **P < 0.01, ***P < 0.001).

According to the design, the AuNTF nanoclusters were expected to capture and convert the overexpressed copper ions in the tumor cells to perform copper-induced chemodynamic therapy, copper-dependent photothermal therapy and copper-deficiency-medicated metastasis inhibition. Therefore, the ROS generation in the tumor cells (4T1 cells) after treatment by using the AuNTF was investigated to demonstrate the copper-induced chemodynamic therapy. The PBS and nanoclusters without modification of FA (AuNT), TPE (AuNF) or NACB (AuTF) were used as control groups. The concentration of all the nanoparticles was 100 μg/mL for the intracellular experiments. As can be seen from Fig. 4a, negligible ROS was detected in the 4T1 cells after treatment using the PBS and AuTF. In contrast, some ROS-positive 4T1 cells were observed in the AuNT group due to the introduction of the NACB segment, which could capture and reduce the overexpressed copper ions to generate cuprous ions, resulting in the cuprous ions-mediated conversion of the excess H_2_O_2_ in the tumor cells into cytotoxic ROS. As expected, the additional introduction of FA (AuNTF and AuNF) further raised the productivity of ROS in the tumor cells due to the enhanced internalization via tumor-targeted delivery, which was regardless of the TPE modification.

We believe the *in situ* production of ROS along with the formation of AuNTF aggregates (photothermal agent) would further damage tumor cells via chemodynamic therapy and photothermal therapy; therefore, the viability of 4T1 cells after treatment by AuNTF with or without NIR irradiation was investigated by using MTT assay, in which the time point for the application of NIR irradiation was selected according to either FITC-medicated fluorescence imaging (8 h after incubation) or AIE-medicated fluorescence imaging (10 h after incubation). The AuNT, AuNF and AuTF with or without NIR irradiation were used as control groups. As can be seen from Fig. 4b, the AuTF group only presented negligible cytotoxicity to 4T1 cells whether with or without NIR radiation. In contrast, the AuNT group exhibited some cytotoxicity to 4T1 cells due to the copper-induced chemodynamic process, which was further enhanced by the FA-mediated tumor-targeted delivery (AuNF group and AuNTF group). As expected, the much lower viability of the tumor cells in these groups was observed after NIR radiation, which could be attributed to the synergistic effect of the chemodynamic therapy and photothermal therapy. Moreover, the AuNTF group with AIE-guided NIR radiation presented the strongest cytotoxicity by far over that with the FITC-guided NIR radiation, indicating that the AuNTF, by combining the AIE property and the various copper-mediated antitumor properties, is able to perform the optimal tumor therapy as designed.

Besides therapeutic efficiency, therapeutic accuracy is also important for tumor treatment. Therefore, the viability of healthy cells (MCF-10A cells) after treatment by AuNTF and NIR irradiation was further investigated by using MTT assay. As can be seen from Fig. 4c, the viability of MCF-10A cells was still maintained at ≤90% after therapy, no matter what time the NIR radiation was applied. This six-times difference in the cytotoxicity between healthy cells and tumor cells after treatment using AuNTF with AIE-guided NIR radiation clearly demonstrated the potential of our nanoclusters to perform precise tumor therapy for clinical application.

Considering the crucial role of copper for tumor migration/invasion and vascularization closely relating to tumor metastasis, the copper-capture performance of our AuNTF was expected to suppress these activities for metastasis inhibition. Therefore, the effects of the AuNTF on the migration and invasion of 4T1 cells were evaluated by using the scratch healing experiment and Transwell cell invasion experiment. The PBS, AuNT, AuNF and AuTF with or without NIR irradiation were used as control groups. As shown in Fig. 4d and e, compared with the groups without the function of copper capture (PBS and AuTF), the migration speed and distance of the 4T1 cells after treatment using the AuNF, AuNT or AuNTF groups were significantly reduced, which could be attributed to the occurrence of NACB (copper ions chelator)-mediated copper deficiency in the tumor cells. Moreover, due to the benefit of tumor-targeting phagocytosis from FA, the efficiency of the AuNTF and AuNF is much higher than that of the AuNT, which was not affected by the modification of TPE (AIE segment). A similar phenomenon was observed in the anti-invasion experiments of these nanostructures in which the AuNTF presented the fewest 4T1 cells through the polycarbonate membrane of the Transwell device (Fig. 4f and g). After demonstrating the ability of the AuNTF against tumor migration and invasion, the effects of the AuNTF on the tube-forming of human umbilical vein endothelial cells (HUVECs) were also evaluated to investigate its anti-vascularization ability. As can be seen from Fig. 4h and i, the tubular networks in the matrix coating plate were significantly reduced as long as the HUVECs were treated using nanostructures with NACB (copper ions chelator) on the surface and the efficiency was further enhanced by the FA-mediated tumor-targeting delivery, leading to the strongest inhibition of vascularization in the AuNTF group. These results clearly indicated the great property of our nanoclusters to inhibit the metastasis of tumor cells via the suppression of tumor motion and the blocking of transfer routes at the same time.

To further investigate the mechanism of the AuNTF against migration and invasion, three representative protein markers of epithelial mesenchymal transition (EMT, a typical process in tumor migration and invasion) in 4T1 cells after treatment by using various formulas were examined via Western blotting analysis. As expected, the AuTF group contributed nothing to the EMT, while the AuNT, AuNF and AuNTF presented the upregulation of E-cadherin expression as well as the downregulation of N-cadherin expression and Vimentin expression, where the efficiency was in the order of AuNTF = AuNF > AuNT, indicating that our AuNTF would effectively reduce the migration and invasion of tumor cells via effective inhibition of its epithelial mesenchyme [52–55] (Fig. 4j and k). This process could be attributed to the Ctr1 (a transmembrane protein responsible for cellular copper uptake) silence once the excess copper was plundered in the tumor cells, which could further inhibit EMT via HIF-1α destabilization, along with Twist and Snail downregulation [56–58]. To prove this process, the expression of Twist and Snail in tumor cells after treatment by using PBS and AuNTF was detected by using Western blotting. As shown in Fig. S14, the AuNTF obviously decreased the expression of Twist and Snail, indicating that the AuNTF could inhibit the EMT process via effective downregulation of Twist and Snail. Moreover, it is worthy to mention that, although the AuNF is able to perform equivalent copper capture to inhibit tumor metastasis similarly to the AuNTF, the relatively low cytotoxicity caused by the photothermal conversion of the AuNF lacking AIE guidance led to less competitiveness than that of AuNTF for tumor therapy, which has been evidenced by the MTT assay presented in Fig. 4b.

More information about the copper-plunder-dependent metastasis inhibition induced by our AuNTF is presented in Figs  S15–S20. As shown in Fig. S15, the copper level in the 4T1 cells dropped dramatically against time after co-incubation with the AuNTF, which was detected by using atomic absorption spectrometry, indicating the strong ability of our AuNTF to grab copper from tumor cells. In contrast, the nanoparticles without the copper chelator (AuTF) caused a negligible change in the level of copper ions even after 12 h of co-incubation. In order to distinguish the change in copper ions and cuprous ions, we further investigated the intracellular Cu^+^ level before and after AuNTF treatment; the typical Cu^+^ indicator (bathocuproine) and UV–Vis spectra were employed according to our previous studies [56,59,60]. The bathocuproine can specifically react with Cu^+^ to form a chromogenic complex, which performed a characteristic absorption at 480 nm. In addition, we could also calculate the level of Cu^2+^ by the difference between the total copper concentration (measured by using atomic absorption spectrometry) and the Cu^+^ concentration (measured by using the bathocuproine and UV–Vis spectra). As can be seen from Fig. S16a and b, the 4T1 cells only contain a small amount of free Cu^+^, which is far less than that of Cu^2+^. After incubation with the AuNTF, these nanoparticles would rapidly capture Cu^2+^ to reduce its level while maintaining the low intracellular concentration of Cu^+^. The resulting AuNTF–copper complex was also observed by using cell lysis and centrifugation, which majorly contains Cu^+^ instead of Cu^2+^ (Fig. S16c and d), indicating that the chelated Cu^2+^ by using our AuNTF had been efficiently converted into Cu^+^ for further chemodynamic therapy as designed. To more directly prove that the AuNTF could reduce the intracellular Cu^2+^ level, we further measured the Cu^2+^ level in 4T1 after 12 h of treatment with the AuNTF by using a Cell Copper Colorimetric Assay Kit with the typical Cu^2+^ indicator DiBr-PAESA [61]. As shown in Fig. S17, the Cu^2+^ level in the 4T1 cells was significantly decreased after AuNTF treatment compared with the PBS group.

More information about the capture and conversion of intracellular copper by our AuNTF are provided by the XPS. In order to perform this experiment, the resulting complex of AuNTF–copper was further collected and incubated under the simulated physiological conditions (PBS solution) for 48 h, then measured by using XPS. It can be seen from Fig. S18 that all the copper ions were converted into cuprous ions after capture, and the cuprous ions were able to stably combine with the AuNTF for a sustained time. These results indicated that the copper ions in the tumor were converted into cuprous ions by our AuNTF, and the cuprous ions would not be used by tumor cells to promote their metastasis due to the formation of the stable complex of AuNTF–copper.

Direct evidence was provided by the invasion and scratch experiment of the 4T1 cells after incubation with the PBS (control group), copper ions, copper ions + AuNTF, cuprous ions and cuprous ions + AuNTF, respectively (Figs S19 and S20). When the copper ions or cuprous ions were added into the culture medium, the tumor proliferated faster than the control group, which resulted in more 4T1 cells through the polycarbonate membrane of the Transwell device. Similar results were obtained from the scratch experiment of groups treated by copper ions or cuprous ions, which showed a much longer distance of tumor migration than that of the PBS group. In contrast, once the AuNTF was added, both tumor invasion and migration were significantly inhibited, even if there were plenty of copper ions or cuprous ions in the culture medium. All these results indicated that, although our AuNTF nanoparticles would effectively capture and convert the copper ions into cuprous ions in tumor cells, the resulting AuNTF–copper complex would not participate in the cell metabolism of copper ions due to the strong binding between the cuprous ions and the AuNTF, resulting in the inhibition of copper-mediated tumor proliferation and metastasis.

### *In vivo* tumor therapy of AuNTF nanoclusters

**Figure 5.**
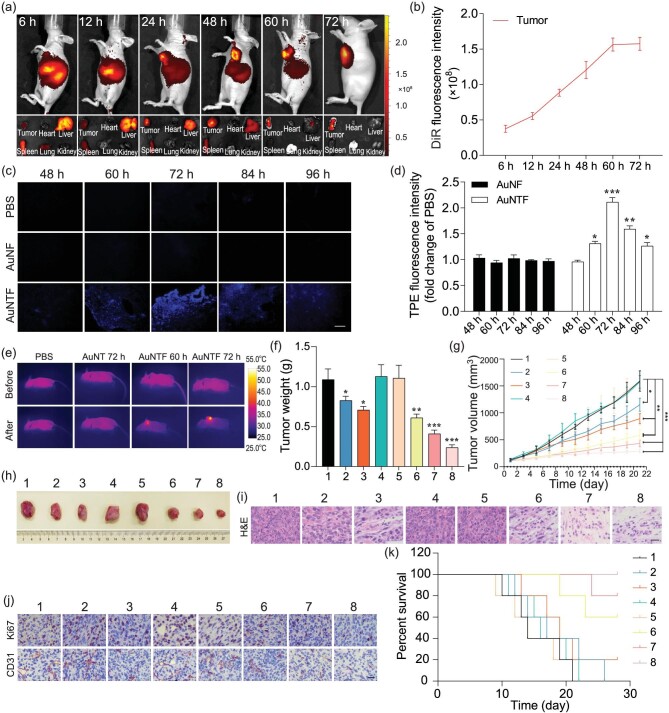
(a) *In vivo* fluorescence images of tumor-bearing mice after injection of DiR-labeled AuNTF nanoparticles at different times (top images), as well as the coresponding *ex vivo* fluorescence images of the tumor and major organs obtained from these mice (bottom images). (b) Quantification of DiR fluorescence intensity in tumor tissue at different injection times according to (a). (c) Fluorescence microscopy images of tumor tissues obtained from tumor-bearing mice after injection of PBS, AuNF and AuNTF for 48, 60, 72, 84 and 96 h. The scale bar is 100 µm. (d) Quantification of AIE fluorescence intensity in tumor tissue according to (c). (e) *In vivo* photothermal images of tumor-bearing mice with treatments of PBS, AuNT and AuNTF before and after 808 nm NIR irradiation. The irradiation was performed at the designated time after the injection of various formulas in which 60 h was selected according to the DiR fluorence guidance and 72 h was selected according to the AIE fluorence guidance. (f) Weights and (g) volumes of the tumor tissues in the tumor-bearing mice after different therapies measured after 21 days. (h) Digital photos, (i) H&E staining as well as (j) Ki 67 and CD 31 staining of the final tumor tissues harvested from tumor-bearing mice after different therapies at Day 21. The scale bar is 100 µm. (k) The survival rate of tumor-bearing mice after different therapies (1: PBS, 2: AuNT, 3: AuNT with NIR irradiation 60 h after injection, 4: AuTF, 5: AuTF with NIR irradiation 60 h after injection, 6: AuNTF, 7: AuNTF with NIR irradiation 60 h after injection, 8: AuNTF with NIR irradiation 72 h after injection). Data are presented as means ± SD (*n* = 5). The laser power was 1.25 W, the concentration was 20 µg/g, the excitation and detection wavelengths of AIE were 350 nm and 398 nm, and the injection method was tail vein injection. The asterisks indicate that the differences between the test and control groups are statistically significant using an ANOVA test (*P < 0.05, **P < 0.01, ***P < 0.001).

After demonstrating the AIE-guided antitumor performance of the AuNTF nanoclusters via copper capture, the copper-activated AIE property, copper-catalysed ROS generation and copper-dependent photothermal conversion at the cellular level, the *in vivo* therapeutic efficacy of the AuNTF nanoclusters was further investigated using 4T1 homograft tumor models. Firstly, the biodistribution of the AuNTF was monitored by using a PerkinElmer IVIS Lumina III In Vivo Imaging System after the injection of DiR (1,1′-dioctadecyl-3,3,3′,3′-tetramethylindotricarbocyanine iodide) labeled AuNTF in tumor-bearing mice. As can be seen from Fig. 5a and b, the AuNTF gradually accumulated in the tumor tissue after injection due to the FA-mediated tumor targeting, the enhanced permeability and retention (EPR) effect, and the enhanced retention of the AuNTF after copper-induced aggregation, which reached a maximum amount in 60 h. Although some AuNTF appeared in the major organs such as the liver and spleen at the beginning of the injection, the rapid metabolism led to a negligible concentration of AuNTF in these organs after 60 h (Fig. S21), which benefited the further tumor-specific therapy. In addition, we further investigated the biodistribution of the copper ions by using an atomic absorption spectrometer after nitrification. As shown in Fig. S22, the copper level in the tumor was gradually enhanced against time and the final amount of copper in the tumor at 72 h was far over that in the other organs, which was very consistent with the *in vivo* fluorescence image of the tumor-bearing mice after the injection of DiR-labeled AuNTF. This result further indicated that the AuNTF would gradually accumulate in the tumor tissue. Moreover, these intratumoral nanoparticles would continuously capture the surrounding copper, which was uninterruptedly supplied from the other part of the body to tumors via blood vessels, resulting in the significantly high level of copper in tumors for chemodynamic therapy as well as the copper-induced aggregation for AIE-guided photothermal therapy.

Considering that copper ions also exist in blood, the risk of AuNTF aggregation in blood resulting in thrombosis was also evaluated. In order to perform this experiment, the level of copper ions in the blood was tested and was ∼0.48 mg/L, as shown in Fig. S23a. As to verify whether the 0.48 mg/L of Cu^2+^ was able to trigger the aggregation of our AuNTF nanoparticles, the size distribution and absorption of the AuNTF nanoclusters with and without the addition of the endogenous concentration of Cu^2+^ in blood (0.48 mg/L) were measured by using dynamic light scattering (DLS) and UV–Vis spectrum. As can be seen from Fig. S23b, only a small size increase from 10 to ∼50 nm was observed after incubation with 0.48 mg/L of Cu^2+^. This tiny size change did not even affect the UV–Vis spectrum of the AuNTF–copper complex, resulting in a similar spectrum to that of the pure AuNTF, which indicated the good dispersal of our AuNTF nanoparticles under the simulated conditions of blood (Fig. S23c). Therefore, we believe that the AuNTF would not aggregate in blood to cause thrombosis due to its high selectivity to the copper ions concentration.

In order to perform the optimal therapy, the clear display of the copper-dependent aggregation process of the AuNTF in tumor cells is much more important than the imaging of its accumulation in tumor tissue. Therefore, the tumor tissue was collected and investigated by using a fluorescence microscope to determine the copper-dependent aggregation of the AuNTF (origin of all theranostics) through the appearance of the TPE performance. The PBS and AuNF served as control groups. As can be seen from Fig. 5c and d, the obvious blue AIE fluorescence gradually appeared in, and only in, the AuNTF group after injection, which reached the maximum at 72 h. This phenomenon not only clearly presented the activation of the AuNTF in real time, but also precisely demonstrated that the best time point for the introduction of NIR radiation to perform optimal photothermal therapy is 72 h after injection, which has been verified by the thermography (Fig. 5e).

To further assess the therapeutic efficacy, the tumor tissue after treatment by the AuNTF with and without NIR radiation was monitored for 3 weeks. The PBS, AuNT and AuTF nanoclusters were employed as control groups. The concentration of all the nanoparticles was 10 mg/kg for the animal experiments. A schematic illustration of the *in vivo* antitumor procedure is shown in Fig. S24. As can be seen from Fig. 5f–h, the tumor tissue significantly grew after treatment using PBS and AuTF whether with or without NIR radiation due to the lack of a copper ions chelator to trigger the therapeutic processes, which resulted in tumor tissue with highest volume and weight. In contrast, the AuNT group presented obvious inhibition of tumor growth due to the copper plunder and copper-triggered chemodynamic therapy in the tumor cells, which could be further enhanced by the additional photothermal therapy (AuNT with NIR radiation) and tumor-targeting delivery (AuNTF). Moreover, the AuNTF with NIR radiation performed the strongest tumor inhibition, which could be attributed to the synergistic effect of the tumor-targeting delivery, copper plunder, photothermal therapy and chemodynamic therapy. As expected, the AuNTF with NIR radiation at the correct time point guided by AIE performed with the best efficiency.

These tumor tissues after 3-week treatments were further collected and measured by using histological analyses including hematoxylin and eosin (H&E) as well as immunohistochemical staining of proliferative marker (Ki 67) and angiogenesis marker (CD 31). Compared with the tumors from the PBS and AuNT groups, tumors from the therapeutic groups (AuNF and AuNTF nanoparticles with and without 808 nm irradiation) all showed obvious tumor damage (HE staining), proliferation inhibition (Ki 67 staining) and vascularization suppression (CD 31 staining), in which the AuNTF under NIR radiation at the correct time point guided by AIE presented the optimal antitumor performance with the largest area of tumor disintegration as well as the strongest downregulation of Ki 67 and CD 31 (Fig. 5i and j).

Moreover, as a critical parameter for evaluating the potential for clinical applications, the survival rates of the tumor-bearing mice in the different groups were also measured. As can be seen from Fig. 5k, mice after treatment with the AuTF all died in ∼22 days whether with or without NIR irradiation, which is similar to the results of the PBS group. The AuNT treatment extended the life cycles of these mice to 26 days, which could be promoted by additional NIR radiation to 28 days with a 20% survival rate. In addition, the AuNTF treatment with or without NIR radiation both presented further enhancement of the survival rate of tumor-bearing mice, which resulted in survival rates of 60% and 80%, respectively. As expected, the group of AuNTF with AIE-guided NIR radiation had the best therapeutic effect in which all the tumor-bearing mice survived for 28 days after therapy, suggesting that our AuNTF could effectively inhibit the primary tumor to prolong the survival period of the tumor-bearing mice via a series of synergistic theranostics.

**Figure 6.**
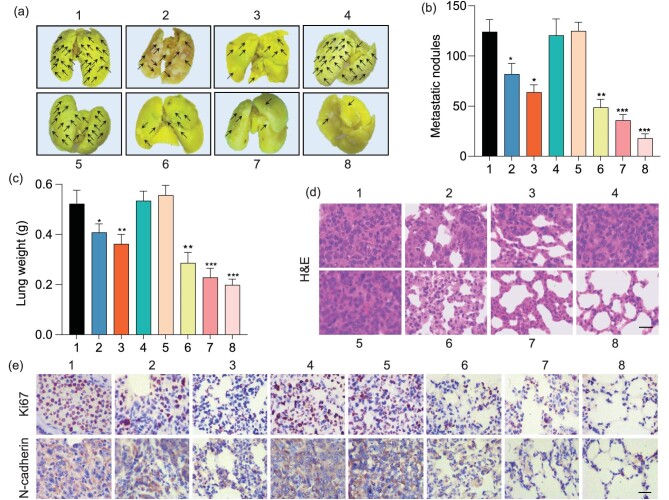
(a) Representative photographs of whole lungs obtained from tumor-bearing mice after various treatments. (b) Quantification of metastatic nodules in lungs of mice in each treatment group. (c) Weights and (d) H&E staining of lungs obtained from tumor-bearing mice after various treatments. The scale bar is 100 μm. (e) Immunohistochemical images showing the expression of Ki 67 and N-cadherin in pulmonary metastatic tumor tissues after various treatments. The scale bar is 100 μm. 1: PBS, 2: AuNT, 3: AuNT with NIR irradiation 60 h after injection, 4: AuTF, 5: AuTF with NIR irradiation 60 h after injection, 6: AuNTF, 7: AuNTF with NIR irradiation 60 h after injection, 8: AuNTF with NIR irradiation 72 h after injection. Data are presented as means ± SD (*n* = 5). The asterisks indicate that differences between the test and control groups are statistically significant using an ANOVA test (*P < 0.05, **P < 0.01, ***P < 0.001).

Considering that metastasis is another key lethal factor of malignant tumors besides the growth of the primary tumor, the anti-metastasis performance of the AuNTF was further assessed by evaluating the lungs of tumor-bearing mice after various treatments. As shown in Fig. 6a and b, the lungs from the PBS group and the AuTF group were filled with metastatic nodules whether with or without NIR irradiation. The metastasis could be significantly suppressed by the therapeutic groups in which the AuNTF with AIE-guided NIR radiation performed with the highest efficiency as expected, resulting in negligible nodules in the lung tissue after therapy. This effective metastasis inhibition was further verified by the well-maintained structure of the lungs, which is clearly presented in Fig. 6c and d through the weight measurement of the lung tissue and H&E staining of the lung tissue sections. In addition, immunohistochemical staining was also utilized to detect the expression of Ki 67 and N-cadherin in the lung tissues, which are the key markers for evaluating tumor proliferation and invasion, respectively. As can be seen from Fig. 6e, the significant downregulation of Ki 67 and N-cadherin expression was observed in the AuNTF-treated group with AIE-guided NIR irradiation. These results suggested that the treatment of AuNTF with AIE-guided NIR irradiation not only dramatically reduced the metastatic nodules in the lung tissue, but also significantly weakened the proliferative capacity and invasiveness of the tumor cells in these nodules, indicating the excellent anti-metastasis ability of the AuNTF *in vivo*.

Besides the therapeutic efficiency, the therapeutic safety of AuNTF treatments was also evaluated by measuring the spleens weight and body weight of tumor-bearing mice after various treatments. As shown in Figs S25 and S26, there was no visible difference between these therapeutic groups and the PBS group, demonstrating the high bio-safety of this strategy right to the end of therapy. In addition, no significant difference was observed in the H&E staining of major organs (heart, liver, spleen, kidney) between the treatments of PBS and AuNTF (Fig. S27), which further indicated the high safety of our strategy for tumor therapy.

## CONCLUSIONS

In this study, gold nanoclusters (AuNTF) co-modified with TPE (AIE segment), NACB (copper ions chelator) and FA (targeting agent) were fabricated to utilize and manipulate the overexpressed copper ions in tumor cells for effective and accurate tumor theranostics via intracellular copper plunder, copper-mediated AIE imaging, copper-induced chemodynamic therapy and copper-dependent photothermal therapy, in which the AIE imaging not only monitored the whole therapeutic process in real time, but was also used to guide the photothermal therapy for optimal therapeutic efficiency. It was confirmed that AuNTF nanoclusters would accumulate in the tumor tissue through FA-mediated tumor targeting as well as the EPR effect. After endocytosis, the AuNTF nanoclusters would chelate with the excessive copper ions in tumor cells and significantly reduce the intracellular concentration of copper ions, resulting in the effective inhibition of tumor metastasis. In the meantime, the chelated copper ions were simultaneously reduced to cuprous ions, which further catalysed the excess H_2_O_2_ in the tumor cells to generate the highly cytotoxic ROS, leading to additional copper-induced chemodynamic therapy. Moreover, the chelation also caused the aggregation of AuNTF nanoclusters, which not only formed a photothermal agent *in situ* with long-term retention for sustained photothermal therapy under NIR radiation, but also activated the AIE property for fluorescence imaging to monitor all the therapeutic processes. This copper-dependent activation of AIE imaging along with AuNTF aggregation further provided a signal to indicate the correct time point of NIR radiation for effective and accurate photothermal therapy. Both cellular and animal experiments exhibited that the tumor-selective photothermal therapy and chemodynamic therapy inflicted significant damage on the primary tumor, while the copper deficiency further hindered the metastasis of tumor cells.

This strategy not only takes advantage of the tumor-specific environment for synergistic therapies, but also, for the first time, introduces AIE agents for real-time imaging of the location and aggregation state of gold nanoparticles (AuNPs), which are two critical issues for the efficiency of photothermal therapy based on AuNPs. Therefore, we are able to perform the infrared irradiation at the most appropriate area and time point according to the location and the aggregation state of AuNPs, which would result in the optimal efficiency of photothermal therapy and minimum damage to the surrounding normal tissue. The much higher efficiency and accuracy of our strategy than those of the conventional photothermal therapy based on AuNPs have been clearly verified both *in vivo* and *in vitro* in the study, which we believe would significantly promote the development of photothermal therapy against various malignant tumors.

## Supplementary Material

nwae113_Supplemental_File

## References

[1] Sung H, Ferlay J, Siegel RL et al. Global cancer statistics 2020: GLOBOCAN estimates of incidence and mortality worldwide for 36 cancers in 185 countries. CA Cancer J Clin 2021; 71: 209–49. 10.3322/caac.21660 33538338

[2] Miller KD, Nogueira L, Devasia T et al. Cancer treatment and survivorship statistics, 2022. CA Cancer J Clin 2022; 72: 409–36. 10.3322/caac.21731 35736631

[3] Kuderer NM, Desai A, Lustberg MB et al. Mitigating acute chemotherapy-associated adverse events in patients with cancer. Nat Rev Clin Oncol 2022; 19: 681–97. 10.1038/s41571-022-00685-3 36221000

[4] Mahvi DA, Liu R, Grinstaff MW et al. Local cancer recurrence: the realities, challenges, and opportunities for new therapies. CA Cancer J Clin 2018; 68: 488–505. 10.3322/caac.21498 30328620 PMC6239861

[5] Alsina M, Arrazubi V, Diez M et al. Current developments in gastric cancer: from molecular profiling to treatment strategy. Nat Rev Gastro Hepat 2023; 20: 155–70. 10.1038/s41575-022-00703-w 36344677

[6] Nam J, Son S, Park KS et al. Cancer nanomedicine for combination cancer immunotherapy. Nat Rev Mater 2019; 4: 398–414. 10.1038/s41578-019-0108-1

[7] Jia C, Guo Y, Wu F. Chemodynamic therapy via Fenton and Fenton-like nanomaterials: strategies and recent advances. Small 2022; 18: 2103868. 10.1002/smll.202103868 34729913

[8] Tang Z, Liu Y, He M et al. Chemodynamic therapy: tumour microenvironment-mediated fenton and Fenton-like reactions. Angew Chem Int Ed 2019; 58: 946–56. 10.1002/anie.201805664 30048028

[9] Li X, Lovell JF, Yoon J et al. Clinical development and potential of photothermal and photodynamic therapies for cancer. Nat Rev Clin Oncol 2020; 17: 657–74. 10.1038/s41571-020-0410-2 32699309

[10] Liu S, Pan X, Liu H. Two-dimensional nanomaterials for photothermal therapy. Angew Chem Int Ed 2020; 59: 5890–900. 10.1002/anie.201911477 32017308

[11] Xie J, Fan T, Kim JH et al. Emetine-loaded black phosphorus hydrogel sensitizes tumor to photothermal therapy through inhibition of stress granule formation. Adv Funct Mater 2020; 30: 2003891. 10.1002/adfm.202003891

[12] Liu B, Sun J, Zhu J et al. Injectable and NIR-responsive DNA-inorganic hybrid hydrogels with outstanding photothermal therapy. Adv Mater 2020; 32: 2004460. 10.1002/adma.202004460 32830376

[13] Zhang H, Cui W, Qu X et al. Photothermal-responsive nanosized hybrid polymersome as versatile therapeutics codelivery nanovehicle for effective tumor suppression. Proc Natl Acad Sci USA 2019; 116: 7744–9. 10.1073/pnas.1817251116 30926671 PMC6475426

[14] Bai G, Yuan P, Cai B et al. Stimuli-responsive scaffold for breast cancer treatment combining accurate photothermal therapy and adipose tissue regeneration. Adv Funct Mater 2019; 29: 1904401. 10.1002/adfm.201904401

[15] Yang Z, He W, Zheng H et al. One-pot synthesis of albumin-gadolinium stabilized polypyrrole nanotheranostic agent for magnetic resonance imaging guided photothermal therapy. Biomaterials 2018; 161: 1–10. 10.1016/j.biomaterials.2018.01.026 29421546

[16] Lin R, Yu W, Chen X et al. Self-propelled micro/nanomotors for tumor targeting delivery and therapy. Adv Healthc Mater 2021; 10: 2001212. 10.1002/adhm.202001212 32975892

[17] Kang H, Rho S, Stiles WR et al. Size-dependent EPR effect of polymeric nanoparticles on tumor targeting. Adv Healthc Mater 2020; 9: 1901223. 10.1002/adhm.201901223 PMC7224408 31794153

[18] He J, Li C, Ding L et al. Tumor targeting strategies of smart fluorescent nanoparticles and their applications in cancer diagnosis and treatment. Adv Mater 2019; 31: 1902409. 10.1002/adma.201902409 31369176

[19] Du B, Ma C, Ding G et al. Cooperative strategies for enhancing performance of photothermal therapy (PTT) agent: optimizing its photothermal conversion and cell internalization ability. Small 2017; 13: 1603275. 10.1002/smll.201603275 28112858

[20] Jaque D, Maestro LM, Rosal B et al. Nanoparticles for photothermal therapies. Nanoscale 2014; 6: 9494–530. 10.1039/C4NR00708E 25030381

[21] Tian Q, Li Y, Jiang S et al. Tumor pH-responsive albumin/polyaniline assemblies for amplified photoacoustic imaging and augmented photothermal therapy. Small 2019; 15: 1902926. 10.1002/smll.201902926 31448572

[22] Wang X, Yang T, Yu Z et al. Intelligent gold nanoparticles with oncogenic microRNA-dependent activities to manipulate tumorigenic environments for synergistic tumor therapy. Adv Mater 2022; 34: 2110219. 10.1002/adma.202110219 35170096

[23] Guo X, Su Q, Liu T et al. Intelligent gold nanoparticles for synergistic tumor treatment via intracellular Ca^2+^ regulation and resulting on-demand photothermal therapy. Chem Eng J 2022; 433: 133850. 10.1016/j.cej.2021.133850

[24] Jin R, Liu Z, Bai Y et al. Core-satellite mesoporous silica-gold nanotheranostics for biological stimuli triggered multimodal cancer therapy. Adv Funct Mater 2018; 28: 1801961. 10.1002/adfm.201801961

[25] Liu T, Jin R, Yuan P et al. Intracellular enzyme-triggered assembly of amino acid-modified gold nanoparticles for accurate cancer therapy with multimode. ACS Appl Mater Inter 2019; 11: 28621–30. 10.1021/acsami.9b05943 31293148

[26] Heerboth S, Housman G, Leary M et al. EMT and tumor metastasis. Clin Transl Oncol 2015; 4: 6. 10.1186/s40169-015-0048-3 PMC4385028 25852822

[27] Quail DF, Joyce JA. Microenvironmental regulation of tumor progression and metastasis. Nat Med 2013; 19: 1423–37. 10.1038/nm.3394 24202395 PMC3954707

[28] Steeg PS . Tumor metastasis: mechanistic insights and clinical challenges. Nat Med 2006; 12: 895–904. 10.1038/nm1469 16892035

[29] Fan X, Li Y, Feng Z et al. Nanoprobes-assisted multichannel NIR-II fluorescence imaging-guided resection and photothermal ablation of lymph nodes. Adv Sci 2021; 8: 2003972. 10.1002/advs.202003972 PMC8097375 33977058

[30] Ye F, Huang W, Li C et al. Near-infrared fluorescence/photoacoustic agent with an intensifying optical performance for imaging-guided effective photothermal therapy. Adv Ther 2020; 3: 2000170. 10.1002/adtp.202000170

[31] Dai J, Wu M, Wang Q et al. Red blood cell membrane-camouflaged nanoparticles loaded with AIEgen and poly(I : C) for enhanced tumoral photodynamic-immunotherapy. Natl Sci Rev 2021; 8: nwab039. 10.1093/nsr/nwab039 34691671 PMC8288176

[32] Yu CYY, Xu H, Ji S et al. Mitochondrion-anchoring photosensitizer with aggregation-induced emission characteristics synergistically boosts the radiosensitivity of cancer cells to ionizing radiation. Adv Mater 2017; 29: 1606167. 10.1002/adma.201606167 28195448

[33] Norako ME, Greaney MJ, Brutchey RL. Synthesis and characterization of wurtzite-phase copper tin selenide nanocrystals. J Am Chem Soc 2012; 134: 23–6. 10.1021/ja206929s 22148639

[34] Kang M, Zhang Z, Xu W et al. Good steel used in the blade: well-tailored type-I photosensitizers with aggregation-induced emission characteristics for precise nuclear targeting photodynamic therapy. Adv Sci 2021; 8: 2100524. 10.1002/advs.202100524 PMC8292883 34021726

[35] Zhang Z, Xu W, Kang M et al. An all-round athlete on the track of phototheranostics: subtly regulating the balance between radiative and nonradiative decays for multimodal imaging-guided synergistic therapy. Adv Mater 2020; 32: 2003210. 10.1002/adma.202003210 32696561

[36] Wang M, Yan D, Wang M et al. A versatile 980 nm absorbing aggregation-induced emission luminogen for NIR-II imaging-guided synergistic photo-immunotherapy against advanced pancreatic cancer. Adv Funct Mater 2022; 32: 2205371. 10.1002/adfm.202205371

[37] Chen X, Gao H, Deng Y et al. Supramolecular aggregation-induced emission nanodots with programmed tumor microenvironment responsiveness for image-guided orthotopic pancreatic cancer therapy. ACS Nano 2020; 14: 5121–34. 10.1021/acsnano.0c02197 32283914

[38] Ge EJ, Bush AI, Casini A et al. Connecting copper and cancer: from transition metal signalling to metalloplasia. Nat Rev Cancer 2022; 22: 102–13. 10.1038/s41568-021-00417-2 34764459 PMC8810673

[39] Shao S, Wang K, Xu R et al. A non-cytotoxic dendrimer with innate and potent anticancer and anti-metastatic activities. Nat Biomed Eng 2017; 1: 745–57. 10.1038/s41551-017-0130-9 31015667

[40] Zhao P, Li H, Bu W. A forward vision for chemodynamic therapy: issues and opportunities. Angew Chem Int Ed 2023; 62: 202210415. 10.1002/anie.202210415 36650984

[41] Ma B, Wang S, Liu F et al. Self-assembled copper-amino acid nanoparticles for in situ glutathione ‘AND’ H_2_O_2_ sequentially triggered chemodynamic therapy. J Am Chem Soc 2019; 141: 849–57. 10.1021/jacs.8b08714 30541274

[42] Tie Y, Zheng H, He Z et al. Targeting folate receptor beta positive tumor-associated macrophages in lung cancer with a folate-modified liposomal complex. Sig Transduct Target Ther 2020; 5: 6. 10.1038/s41392-020-0115-0 PMC6976681 32296026

[43] Wang D, Wu SY, Li HP et al. Synthesis and characterization of copper complexes with the N-(2,6-diisopropylphenyl)-N′-acylthiourea ligands. Eur J Inorg Chem 2017; 2017: 1406–13. 10.1002/ejic.201601451

[44] Gao S, Jin Y, Ge K et al. Self-supply of O_2_ and H_2_O_2_ by a nanocatalytic medicine to enhance combined chemo/chemodynamic therapy. Adv Sci 2019; 6: 1902137. 10.1002/advs.201902137 PMC6918120 31871871

[45] Xu Y, Liu S, Zeng L et al. An Enzyme-engineered nonporous copper(I) coordination polymer nanoplatform for cuproptosis-based synergistic cancer therapy. Adv Mater 2022; 34: 2204733. 10.1002/adma.202204733 36054475

[46] Xiao P, Shen Z, Wang D et al. Precise molecular engineering of type I photosensitizers with near-infrared aggregation-induced emission for image-guided photodynamic killing of multidrug-resistant bacteria. Adv Sci 2022; 9: 2104079. 10.1002/advs.202104079 PMC8844491 34927383

[47] Yu X, Zhang Y, Yang X et al. Bonsai-inspired AIE nanohybrid photosensitizer based on vermiculite nanosheets for ferroptosis-assisted oxygen self-sufficient photodynamic cancer therapy. Nano Today 2022; 44: 101477. 10.1016/j.nantod.2022.101477

[48] Li Z, Ji X, Xie H et al. Aggregation-induced emission-active gels: fabrications, functions, and applications. Adv Mater 2021; 33: 2100021. 10.1002/adma.202100021 34216407

[49] Davis DA, Branca AA, Pallenberg AJ et al. Inhibition of the human immunodeficiency virus-1 protease and human immunodeficiency virus-1 replication by bathocuproine disulfonic acid Cu^+^. Arch Biochem Biophys 1995; 322: 127–34. 10.1006/abbi.1995.1444 7574666

[50] Fu J, Shao Y, Wang L et al. Lysosome-controlled efficient ROS overproduction against cancer cells with a high pH-responsive catalytic nanosystem. Nanoscale 2015; 7: 7275–83. 10.1039/C5NR00706B 25813671

[51] Mei L, Ma D, Gao Q et al. Glucose-responsive cascaded nanocatalytic reactor with self-modulation of the tumor microenvironment for enhanced chemo-catalytic therapy. Mater Horiz 2020; 7: 1834–44. 10.1039/D0MH00105H

[52] Fan JQ, Li Y, Wei Z et al. Binding-induced fibrillogenesis peptides recognize and block intracellular vimentin skeletonization against breast cancer. Nano Lett 2021; 21: 6202–10. 10.1021/acs.nanolett.1c01950 34259530

[53] Beijnum JRV, Huijbers EJM, Loon KV et al. Extracellular vimentin mimics VEGF and is a target for anti-angiogenic immunotherapy. Nat Commun 2022; 13: 2842. 10.1038/s41467-022-30063-7 35606362 PMC9126915

[54] Schipper K, Seinstra D, Drenth AP et al. Rebalancing of actomyosin contractility enables mammary tumor formation upon loss of E-cadherin. Nat Commun 2019; 10: 3800. 10.1038/s41467-019-11716-6 31444332 PMC6707221

[55] Gloerich M, Bianchini JM, Siemers KA et al. Cell division orientation is coupled to cell-cell adhesion by the E-cadherin/LGN complex. Nat Commun 2017; 8: 13996. 10.1038/ncomms13996 28045117 PMC5216124

[56] Liu J, Yang T, Zhang H et al. Intelligent nanoreactor coupling tumor microenvironment manipulation and H_2_O_2_-dependent photothermal-chemodynamic therapy for accurate treatment of primary and metastatic tumors. Bioact Mater 2024; 34: 354–65. 38269307 10.1016/j.bioactmat.2023.12.028 PMC10806208

[57] Chang X, Zhu Z, Weng L et al. Selective manipulation of the mitochondria oxidative stress in different cells using intelligent mesoporous silica nanoparticles to activate on-demand immunotherapy for cancer treatment. Small 2023; doi: 10.1002/smll.202307310. 10.1002/smll.202307310 38039438

[58] Li S, Zhang J, Yang H et al. Copper depletion inhibits CoCl_2_-induced aggressive phenotype of MCF-7 cells via downregulation of HIF-1 and inhibition of Snail/Twist-mediated epithelial-mesenchymal transition. Sci Rep 2015; 5: 12410. 10.1038/srep12410 26174737 PMC4502431

[59] Yin T, Yang T, Chen L et al. Intelligent gold nanoparticles for malignant tumor treatment via spontaneous copper manipulation and on-demand photothermal therapy based on copper induced click chemistry. Acta Biomater 2023; 166: 485–95. 10.1016/j.actbio.2023.04.036 37121369

[60] Chen L, Yang T, Weng L et al. Integration of tumor elimination and tissue regeneration via selective manipulation of physiological microenvironments based on intelligent nanocomposite hydrogel for postoperative treatment of malignant melanoma. Adv Funct Mater 2023; 33: 2304394. 10.1002/adfm.202304394

[61] Li D, Shi Z, Liu X et al. Identification and development of a novel risk model based on cuproptosis-associated RNA methylation regulators for predicting prognosis and characterizing immune status in hepatocellular carcinoma. Hepatol Int 2023; 17: 112–30. 10.1007/s12072-022-10460-2 36598701

