# Supplementary material for: Intelligent gold nanocluster for effective treatment of malignant tumor via tumor-specific photothermal–chemodynamic therapy with AIE guidance
Source: Natl Sci Rev. 2024 Mar 22;11(5):nwae113. doi: 10.1093/nsr/nwae113 (PMC11065357; doi:10.1093/nsr/nwae113)
Supplement: nwae113_Supplemental_File [file nwae113_supplemental_file.pdf]

Supporting Information
for
Intelligent gold nanocluster for effective treatment of malignant tumor via tumor-specific photothermal-chemodynamic therapy with AIE guidance

Feng Liu[a,b1], Tianfeng Yang[c1], Xiaowei Chang[b1], Li Chen[b], Cheng Cheng[c], Xiuhong Peng[c], Haihu Liu[a]*, Yanmin Zhang[c]*, Xin Chen[b]*

[a] School of Energy and Power Engineering, Xi'an Jiaotong University, 28 West Xianning Road, Xi'an 710049, China.
[b] Department of Chemical Engineering, Shaanxi Key Laboratory of Energy Chemical Process Intensification, Institute of Polymer Science in Chemical Engineering, School of Chemical Engineering and Technology, Xi’an Jiao Tong University, Xi’an, 710049, China.
[c] School of Pharmacy, Health Science Center, Xi’an Jiaotong University, Xi’an, 710061, China.

[1]These authors contributed equally to this work.
*Corresponding Author (E-mail: chenx2015@xjtu.edu.cn; zhang2008@xjtu.edu.cn; haihu.liu@mail.xjtu.edu.cn)

1. Materials

Gold chloride hydrate ($HAuCl_4 \cdot H_2O$), folic acid (FA, $C_{19}H_{19}N_7O_6$), 1-ethyl-3-(3dimethylaminopropyl) carbodiimide hydrochloride (EDC HCl, 98%) and n-hydroxysuccinimide (NHS, 98%) were purchased from Energy Chemical (Shanghai). Sodium borohydride ($NaBH_4$), 12-Mercaptododecanoic acid ($C_{12}H_{24}O_2S$), 4-(1,2,2-triphenylethenyl)phenol ($C_{24}H_{20}O$), Tetraoctylammonium bromide ($C_{32}H_{68}BrN$), Bathocuproine Disulfonic Acid Disodium Salt (BDADS, $C_{26}H_{18}N_2Na_2O_6S_2$), 3,3’,5,5’-Tetramethylbenzidine (TMB) and Benzoyl isothiocyanate (C_8H_5NOS) were purchased from Aladdin Reagent Co., Ltd. Ethylenediamine anhydrous ($C_2H_8N_2$),

dichloromethane (DCM) and Hydrochloric acid (HCl) were purchased from Sinopharm Chemical Reagent Co., Ltd (Xi'an, China). Aqueous solutions were prepared using ultrapure water from a Milli-Q system (Millipore, USA). All other reagents were of analytical grade and used without purification.

2. Synthesis of thiol functionalized 4-(1,2,2-triphenylethenyl)phenol (TPE-SH, AIE agent)

The TPE-SH was synthesized through the esterification reaction between 12-mercaptododecanoic acid and 4-(1,2,2-triphenylethenyl)phenol. Particularly, 2 mmol 12-mercaptododecanoic acid was dissolved in 5 mL DCM; Then, 2 mmol EDC and 1 mmol NHS were added into the solution; After 30 min catalytic activation, 2 mmol 4-(1,2,2-triphenylethenyl)phenol was added and followed with 24 h reaction with continuous stirring.

3. Synthesis of thiol functionalized N-((2-aminoethy)carbamothioyl) benzamide (NACB-SH, copper chelator)

Ethylenediamine anhydrous (4 mmol) dissolved in 10 mL DCM were slowly dropwise added into benzoyl isothiocyanate solution (2 mmol in 10 mL DCM). The mixture was stirred at room temperature for 4 hours and then acidified to PH=5-6 by dropwise addition of dilute HCl with thorough stirring. The mixture was extracted with DCM, and the organic layer was collected, followed with dehydration by $NaSO_4$. The organic layer was concentrated under reduced pressure to obtain N-((2-aminoethy)carbamothioyl) benzamide. Finally, NACB-SH was obtained through the amidation reaction between 12-Mercaptododecanoic acid (2mmol) and N-((2-aminoethy)carbamothioyl) benzamide (2mmol) under the catalytic activation of EDC and NHS in 10 mL DCM with continuous stirring at room temperature overnight.

4. Synthesis of thiol functionalized folic acid (FA-SH, tumor targeting agent)

The FA-SH was also synthesized through the amidation reaction. The preparation was as follows: 2 mmol 12-mercaptododecanoic was dissolved in 5 mL deionized water; Then, 2 mmol EDC and 1 mmol NHS were added into the solution; After 30 min catalytic activation, 2 mmol folic acid was added and followed by 24 h reaction with continuous stirring.

5. Synthesis of AuNTF Nanoclusters

The synthesis of final AuNTF nanoclusters was as follows: An aqueous solution of gold chloride hydrate (7 mL, 3 mM) was first mixed with a solution of tetraoctylammonium bromide in toluene (13 mL, 5 mM). The two-phase mixture was vigorously stirred until all the gold chloride hydrate was transferred into the organic layer. Then, the organic layer was collected, followed by a slow addition of freshly prepared aqueous solution of sodium borohydride (7 mL, 0.04 M) with vigorous stirring. After stirring for 5 min, TPE-SH (2 μmol), NACB-SH (4 μmol) and FA-SH (1 μmol) were simultaneously added to the organic phase. Next, the mixture was continuously stirred for 3 h until the organic phase faded and gold nanoparticles were transferred into aqueous phase. Finally, the aqueous phase was collected for dialysis, so that gold nanoparticles were obtained which were labeled as AuNTF. Similar to AuNTF, other control groups could be also prepared, including AuF (gold nanoclusters individually functionalized with FA), AuN (gold nanoclusters individually functionalized with NACB), AuT (gold nanoclusters individually functionalized with TPE), AuNT (gold nanoclusters functionalized with NACB and TPE), AuNF (gold nanoclusters functionalized with NACB and FA), and AuTF (gold nanoclusters functionalized with TPE and FA).

6. Measurement of copper dependent photothermal performance of AuNTF nanoclusters

To investigate the photothermal conversion performance of the nanoclusters, the temperatures of AuNTF (1 mL, 1.5 mM) nanoclusters in

different solutions (H_2O with and without Cu^{2+} (0.1 mM)) were measured after irradiation for the same time under 808 nm laser, respectively. The temperature change of the AuNTF solution was recorded using an IR thermal camera (testo 882 Thermal imager, Germany) every 60 s. The solution was exposed to the 808 nm laser for 10 min to reach a steady state of maximum temperature, and then the solution was cooled down to room temperature with the laser shutting off.

7. Measurement of copper dependent cuprous ions generation and ROS production

The cuprous ions generation along with copper capture was measured through chromogenic reaction with BDADS and spectral variation of UV-Vis absorbance. 100 μL of BDADS (10 mM) was added into 900 μL of H_2O as color-substrate solution, and then Cu^{2+} (0.1 mM), Cu^{+} (0.1 mM), AuNTF (1.5 mM), or Cu^{2+} (0.1 mM) + AuNTF (1.5 mM) were added into the solution, respectively. After a given time, the sample was withdrawn and filtrated. Subsequently, the absorbance of supernatant at 480 nm was recorded by UV-Vis absorption spectrophotometer.

To investigate the ROS production *via* decomposition of H_2O_2 catalyzed by the resulting AuNTF-Cu^{+} complex, 100 μL of TMB (8 mM) was added into 900 μL of H_2O as color-substrate solution, and then H_2O_2 (0.1 mM), H_2O_2 (0.1 mM) + Cu^{+} (0.1 mM) or H_2O_2 (0.1 mM) + AuNTF (1.5 mM) + Cu^{2+} (0.1 mM) were added, respectively. After a given time, the sample was withdrawn and filtrated. Subsequently, the absorbance of supernatant at 380 and 660 nm was recorded by UV-Vis absorption spectrophotometer.

To investigate the generation of ROS during the chelation and reduction of Cu^{2+}, 100 μL of TMB (8 mM) was added into 900 μL H_2O as color-substrate solution, and then AuNTF (1.5 mM), Cu^{2+} (0.1 mM) or Cu^{2+} (0.1 mM) + AuNTF (1.5 mM) were added, respectively. After a given time, the sample was withdrawn and filtrated. Subsequently, the absorbance of supernatant at 380 and 660 nm was recorded by UV-Vis absorption spectrophotometer.

8. Characterization

^{1}H NMR spectrum was recorded using an AVANCE III HD 600 MHz spectrometer with dimethyl sulfoxide-d 6 (DMSO-d 6) as the solvent at 293 K. Tetramethylsilane (TMS) was used as the internal standard. Scanning electron microscope (SEM) images and elemental analysis were obtained using a TESCAN MAIA3LMH scanning electron microscope equipped with an energy dispersive spectrometer (EDS). Transmission electron microscopy (TEM) images were recorded on a JEOL JEM-2100Plus transmission electron microscope operated at 200 kV. For the TEM observation, samples were obtained by dropping 10 μL of the solution onto carbon-coated copper grids. All the TEM images were visualized without staining. The infrared (IR) spectra were measured by Nicolet iS50 FT-IR using KBr pellets. The ultraviolet-visible (UV-Vis) spectra were measured with a dilute aqueous solution in a 2 mm thick quartz cell using a Thermo scientific GENESYS 150 spectrophotometer. Fluorescence images were acquired using a Nikon TE20 0 0-U inverted fluorescent microscope equipped with a cooled CCD camera (DS-U1, Nikon Corporation, Japan). Dynamic light scattering (DLS) experiments and Zeta potential measurements were performed using a Zetasizer (Zetasizer Nano ZSE, Malvern, UK) at a temperature of 25 ℃. All the samples were dispersed in deionized water. X-ray photoelectron spectroscopy (XPS) analysis was carried out on a Thermo Fisher ESCALAB Xi^+ spectrometer (all the peaks corrected with reference to the C signal (284.8 eV)). The AIE was monitored by fluorescence microscopy using an Olympus BX51 microscope equipped with a fluorescent lamp; Ex = 350 nm.

9. Cell culture

Human normal mammary epithelial cell MCF-10A and mouse breast cell lines 4T1 were purchased from the Shanghai Institute of Cell Biology, the Chinese Academy of Sciences (Shanghai, China). Human umbilical vein endothelial cells (HUVECs) were obtained from Procell life science

and technology co., Ltd. (Wuhan, China) and cultured under recommended conditions. 4T1 was cultured in RPMI-1640 medium with 10% (v/v) FBS and 1% P/S, while MCF-10A was cultured in DMEM/F12 with 5% (v/v) HS, 20 ng/mL EGF, 0.5 μg/mL Hydrocortison, 10 μg/mL Insulin, 1% NEAA and 1% P/S. All cell lines were incubated at 37 ℃ in a humidified incubator containing 5% CO_2.

10. Cellular uptake

4T1 cells and MCF-10A cells were cultured in 96 well-plates with 180 μL cell suspension per well for 24 h. Then, cells were treated with PBS, FITC-AuNT or FITC-AuNTF for 6 h and 12 h, respectively. For fluorescence microscope study, after rinsed with PBS, cells were fixed with 4% polyformaldehyde. Then, the cell nuclei were stained with DAPI and images were captured by an inverted fluorescence microscope. For flow cytometry analysis, after rinsing cells with PBS, the fluorescence intensity of FITC in the cells were detected and analyzed by flow cytometer (ACEA Novocyte, ACEA, CA, USA).

11. Observation of AIE

At the cellular level, 4T1 cells and MCF-10A cells were cultured in 96 well-plates with 180 μL cell suspension per well for 24 h. Then, PBS, AuNT, AuNF, AuTF or AuNTF were incubated with the cells. After 12 hours of incubation, the cells were rinsed with PBS and fixed with 4% polyformaldehyde. Then, the blue fluorescence of TPE in the cells was observed by an inverted fluorescence microscope.

At the animal level, the tumor tissues in PBS, AuNF and AuNTF groups were collected and incubated in 4% paraformaldehyde and embedded in paraffin. The paraffin samples were sectioned as thick as 5 μm, and then were deparaffinized by xylene. Next, the blue fluorescence of TPE in the tumor cells was observed by an inverted fluorescence microscope.

12. Cell viability assay

MTT method was used to test cell viability. Cells were cultured in 96 well-plates with 180 μL cell suspension per well for 24 h. After treated with different formulas, the cells were maintained in incubator for 48 h. Then the medium was substituted by the serum-free one with 10% MTT solution. DMSO was added after 4-6 h. The plate was measured by a Bio-Rad microplate reader.

13. In vitro AIE imaging

When the AIE imaging was performed in 96-well plate to guide the photothermal therapy of AuNTF nanoparticles, the laser spots of both exciting and NIR lights actually covered the whole well used for co-incubation of AuNTF nanoparticles and tumor cells, and all cells were under radiation. Meanwhile, all the cells were measured by fluorescence microscope and thermal meter to detect the fluorescence intensity and photothermal property. During this process, the AuNTF nanoparticles outside tumor cells would exhibit negligible AIE fluorescence due to its monodisperse state. Once the AuNTF nanoparticles was taken up by tumor cells, the over-expressed copper in tumor cell would induce the aggregation of these nanoparticles, which triggered the AIE fluorescence. With the enhancement of the aggregation degree of AuNTF nanoparticles against incubation time, the intensity of AIE fluorescence would also increase until a peak value. This peak value indicated the maximum aggregation of AuNTF nanoparticles appeared in tumor cells, and the corresponding moment was designed to be the proper time point to perform NIR radiation for optimal photothermal efficiency.

14. ROS detection

The intracellular ROS were measured by DCFH-DA dye. 4T1 and MCF-10A cells were seeded in a 6-well plate. After 24 h, the cells were incubated with PBS, AuNT, AuNF, AuTF, or AuNTF, respectively, under laser irradiation. After 48 h, DCFH-DA working solution was added in the dark. After 20 min incubation, the cells were washed with serum-free medium 3 times and with PBS buffer once. Flow cytometer was used to

detect the fluorescence signals.

15. Scratch assay

After 4T1 cells were grown to 80% confluence, the cell monolayers were scratched using pipette tips and incubated with different formulas in medium containing 3% FBS for 24 h. The scratch distance was measured and the migration rate was then calculated.

16. Transwell migration assay

4T1 cells suspended in complete medium were seeded into the upper chamber and cultured overnight. After treatment with different formulas for 24 h, the medium in upper chamber was replaced with serum-free medium, and the lower chamber was filled with complete medium containing 20% FBS to promote cell migration. The migrated cells on the lower surface were fixed in 95% ethanol for 15 min, stained with 0.1% crystal violet, and counted and photographed using an inverted fluorescence microscope.

17. Tube formation assay

The tube formation assay was performed to determine the effect on angiogenesis *in vitro*. In brief, matrigel was diluted in serum-free RPMI-1640 to 3 mg/ml of the final concentration. 12-well plates were coated with 200 μL/well matrigel and 5 μL/well thrombin (50 U/mL) and incubated at 37 ℃ for 30 min. Following gel formation, HUVEC cells were added to each well and various formulas were applied to each well after indicated time. The plates were incubated at 37 ℃. Images of the formation of capillary tubes were then captured randomly under a microscope.

18. Western blotting

After incubated with PBS, AuNT, AuNF, AuTF or AuNTF, cells were rinsed with PBS and incubated in RIPA buffer on ice for 30 min. Then the product was centrifuged at 4 ℃ for 10 min. After the

measurement of the protein concentrations via a BCA Protein Quantification kit, 5×loading buffer with bromophenol blue was added to the protein supernatant.

After separation on 10% SDS-polyacrylamide gel, the proteins were then transferred from the gel to PVDF membranes. Following incubation with 10% non-fat milk for 2 h, the membranes were treated with primary antibodies (E-cadherin, N-cadherin, Vimentin, GAPDH) for 12 h at 4 ℃. After incubation with secondary antibodies for 1 h at 37 ℃, the membranes were rinsed. Finally, the reactive bands were tested by Tanon 5200 automatic chemiluminescence image analysis system using an ECL kit.

19. Animals and tumor models

Female nude mice (4-6 weeks of age) were used to conduct all the *in vivo* studies. The mice were housed at Laboratory Animal Center of Xi'an Jiaotong University in a specific pathogen-free atmosphere. All the mice studies were performed according to regional authority guidelines.

For the homograft model, the mice were subcutaneously inoculated with 150 μL 4T1 cell suspension (2×10^7 cells/ml) at right flanks. The tumor volume was measured every day as $(A \times B^2)/2$, where A is the longer diameter and B is the shorter diameter. The mice were randomly divided into eight groups (four mice in each group) and treated with indicated groups. After 6 h injection with DIR-labeled AuNTF, the mice were exposed to Caliper IVIS Lumina II System to investigate the *in vivo* tracking of nanoparticles. After 60 h or 72 h post-injection, the tumorous areas of PBS, AuNT or AuNTF treatments with NIR group were exposed to 808 nm NIR irradiation (1 A) for 30 s to investigate the photothermal therapeutic effect *in vivo*. Body weight and tumor volume of each mouse were registered daily. Following 21 days of continuous treatment, the mice were euthanized. The tumors and main organ including lung, heart, liver, spleen and kidney were collected and weighed. The anti-tumor effects of AuNTF were confirmed via hematoxylin and eosin (H&E) staining and immunohistochemistry (IHC) staining.

The *in vivo* antitumor activity was further investigated for survival. Another eight groups with 5 mice per group were monitored every 2 days until the mice were either naturally died or were sacrificed when the tumor volume grew to 2000 mm^3 for survival analysis, according to the animal ethical requirement.

For the metastasis model, each mouse was injected to the tail veins with 200 μL 4T1 cell suspension (1×10^7 cells/mL). After 7 days, mice were randomly divided into eight groups (three mice in each group) and treated with indicated groups. After 21 days of continuous treatment, the mice were sacrificed. The lung tissues were collected and weighed for *in vivo* tumor invasion and metastasis analysis. Metastatic lung nodules were counted and further confirmed via H&E staining and IHC staining.

20. H&E staining and IHC assay

The tumor and lung tissues were incubated in 4% paraformaldehyde and embedded in paraffin. The paraffin samples were sectioned as thick as 5 μm, and then were deparaffinized by xylene. Then, the samples were stained with hematoxylin for 10 min. After treatment with hydrochloric acid alcohol solution and ammonium hydroxide each for 30 seconds, the samples were then stained with eosin for 3 min. The increased concentration of alcohol was used to dehydrate the sections. Then the sections were treated with xylene three times each for 3 min. Finally, neutral balsam was used for section mounting.

The tumor and lung tissues were embedded in paraffin were deparaffinized as mentioned above. After dehydration in a decreased gradient of ethanol, the samples were then incubated with citrate buffer and immersed in 3% H_2O_2 solution for 10 min. Thereafter, the corresponding primary antibodies were applied on the samples at 4℃ overnight. Following incubation with the HRP-conjugated secondary antibodies, the sections were incubated with diaminobenzidine and re-dyed with hematoxylin for capturing images.

21. Statistical analysis

All results are representative of data generated in three independent experiments. All numeric values were expressed as the mean ± SD. For multiple comparisons, statistical analysis was performed using one-way ANOVA followed by a Bonferroni post-test. Data analysis was performed using SPSS 18.0 software and considered statistically significant at $p <$ 0.05.

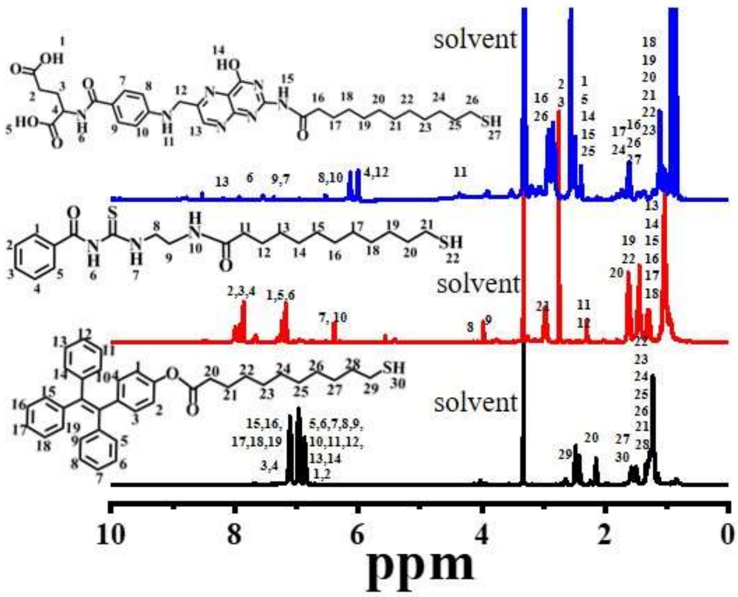

Figure S1. Nuclear magnetic resonance spectroscopy (^{1}H-NMR) of TPE-SH (black curve), NACB-SH (red curve) and FA-SH (blue curve).

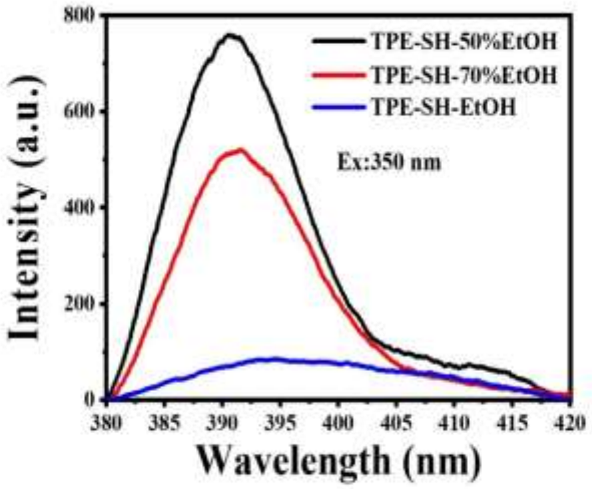

Figure S2. Fluorescence spectra of TPE-SH in water/ethanol solutions

with different ratios, indicating that the aggregation induced emission (AIE) of TPE-SH conformed to the design.

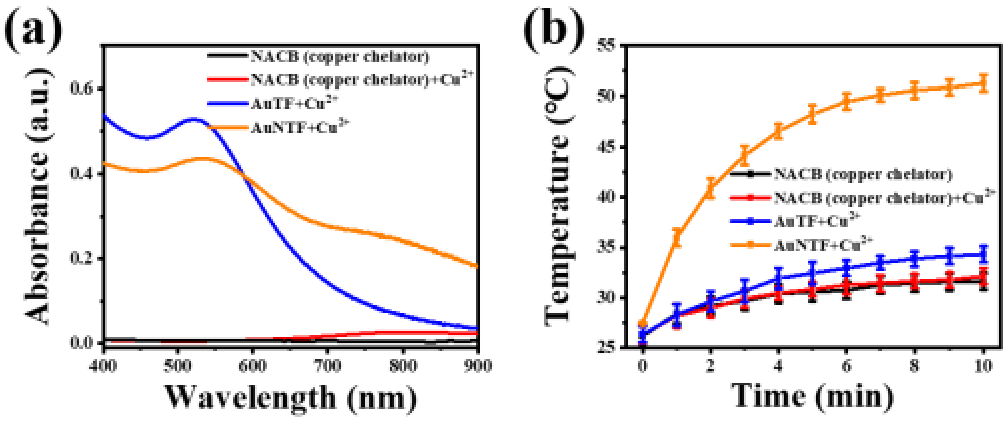

Figure S3. (a) UV-Vis spectra and **(b)** photothermal curves of NACB (copper ions chelator) with and without Cu^{2+} as well as AuTF and AuNTF with Cu^{2+}.

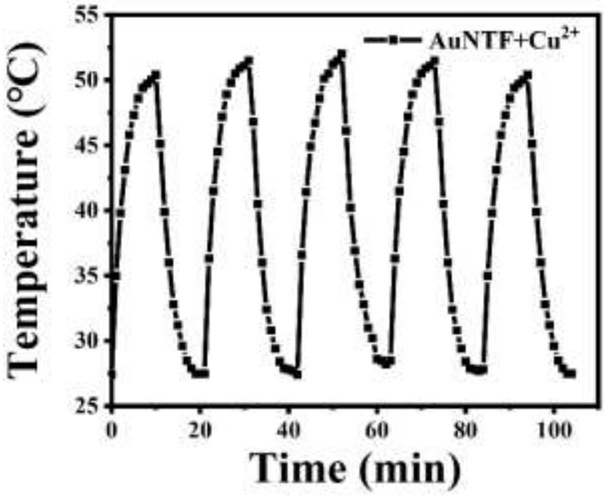

Figure S4. Thermal cycles of AuNTF nanoclusters in solution with Cu^{2+} upon irradiation of 808 nm laser.

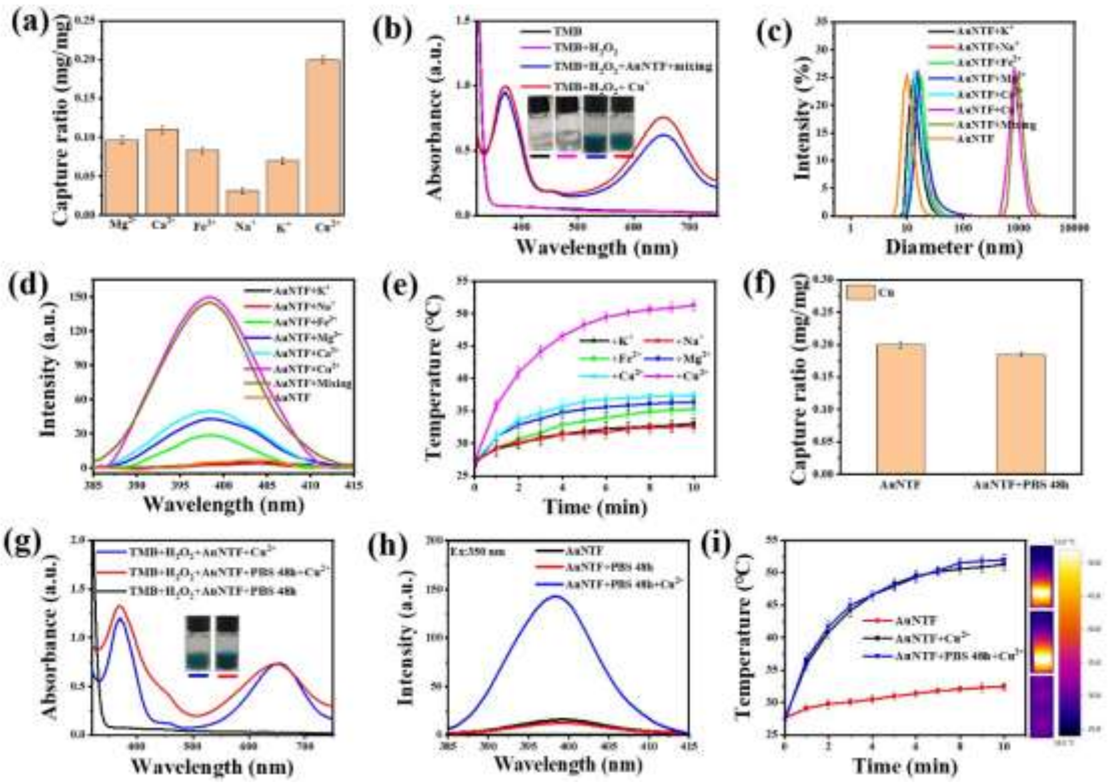

Figure S5. (a) Copper-selective capture properties of AuNTF nanoclusters determined by atomic absorption spectrometry. **(b)** UV-Vis spectra and photographs of TMB solution with and without H_2O_2, H_2O_2+Cu^+ as well as H_2O_2+AuNTF+mixed ions (K^+, Ca^{2+}, Na^+, Mg^{2+}, Fe^{2+} and Cu^{2+}) indicated that the AuNTF would selectively capture and reduce copper from mixed ions to gradually produce cuprous ions and ROS in solution containing H_2O_2 for chemodynamic therapy. **(c-e)** DLS **(c)**, fluorescence spectra **(d)** and photothermal curves **(e)** of AuNTF nanoclusters in solution containing K^+, Ca^{2+}, Na^+, Mg^{2+}, Fe^{2+}, Cu^{2+} or mixed ions indicated that the aggregration, aggregation induced emission (AIE) property and photothermal property of AuNTF nanoclusters could only be triggered by copper ions regardless with the interference of other ions. **(f)** Copper capture properties of AuNTF nanoclusters before and after 48 h incubation in PBS determined by atomic absorption spectrometry. **(g-i)** Copper dependent ROS gneration property **(g)**, AIE property **(h)** and photothermal property **(j)** of AuNTF nanoclusters before and after 48 h incubation in PBS determined by UV-Vis spectra, fluorescence spectra and photothermal curves.

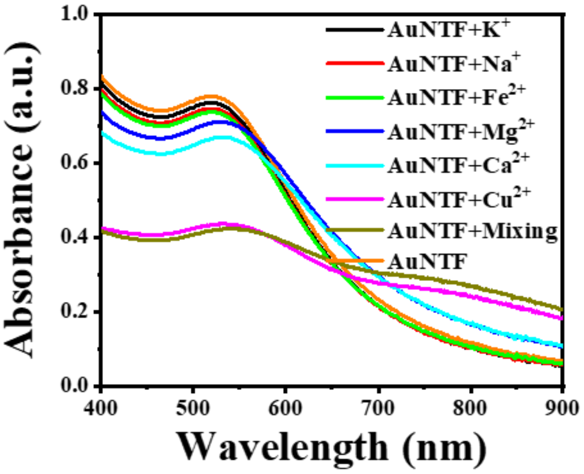

Figure S6. UV-Vis spectra of AuNTF nanoclusters in solution with and without K^{+}, Ca^{2+}, Na^{+}, Mg^{2+}, Fe^{2+}, Cu^{2+} or mixed ions.

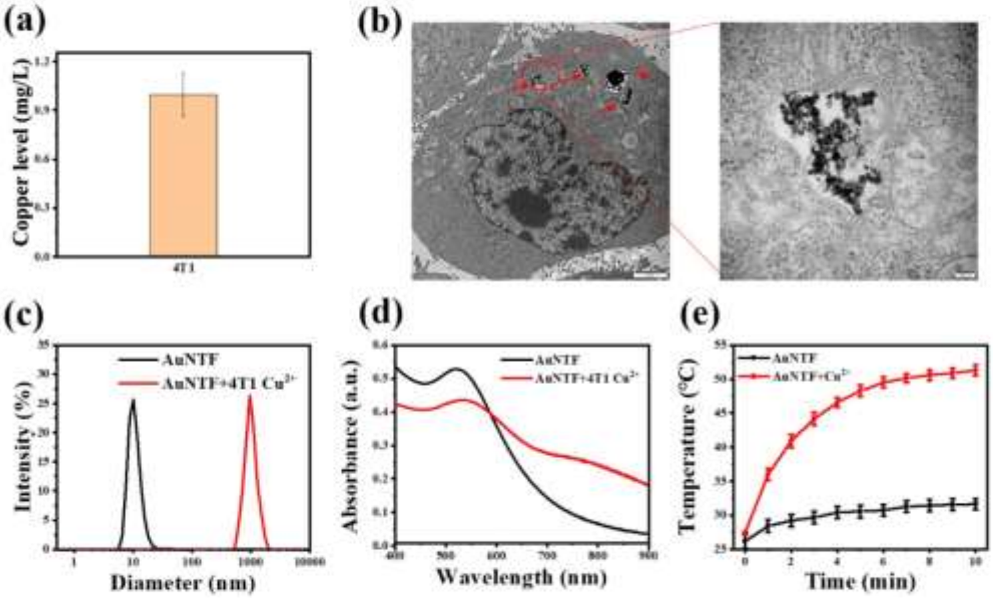

Figure S7. (a) The concentration of copper ions in 4T1 cells. **(b)** Transmission electron microscope (TEM) images of 4T1 cell after incubation with AuNTF for 12 h. The amplified image presented the AuNTF aggregates in 4T1 cell. **(c)** The Dynamic Light Scattering (DLS), **(d)** UV-Vis spectrum and **(e)** photothermal curves of AuNTF nanoparticles with and without addition of the endogenous concentration of Cu^{2+} in 4T1 cells (1 mg/L).

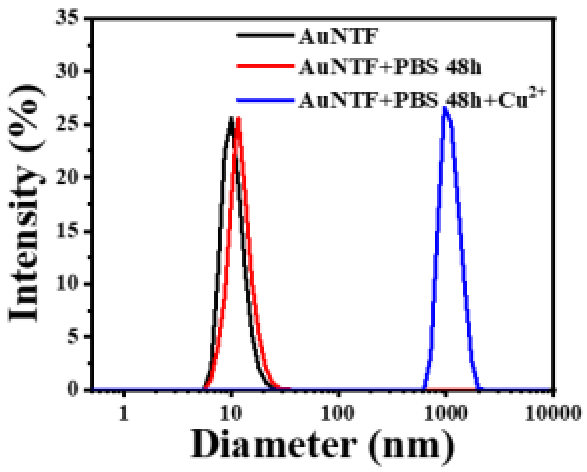

Figure S8. Size distribution of AuNTF nanoclusters before and after 48 h incubation in PBS solution as well as the size distribution of AuNTF nanoclusters after 48 h incubation in PBS solution followed with addition of Cu^{2+}

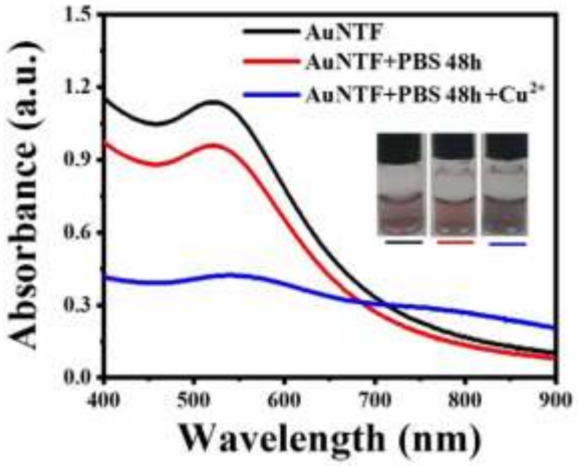

Figure S9. UV-Vis spectra and photographs of AuNTF nanoclusters before and after 48 h incubation in PBS solution as well as the UV-Vis spectra and photographs of AuNTF nanoclusters after 48 h incubation in PBS solution followed with addition of Cu^{2+}.

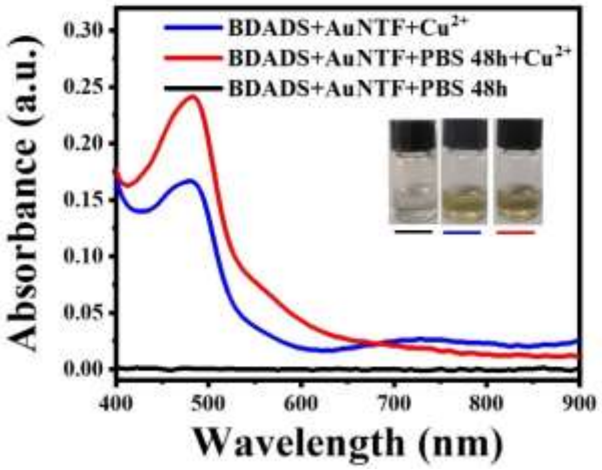

Figure S10. Cuprous ions generation property of AuNTF nanoclusters during copper capture before and after 48 h incubation in PBS determined by UV-Vis spectra and cuprous ions indicator (BDADS).

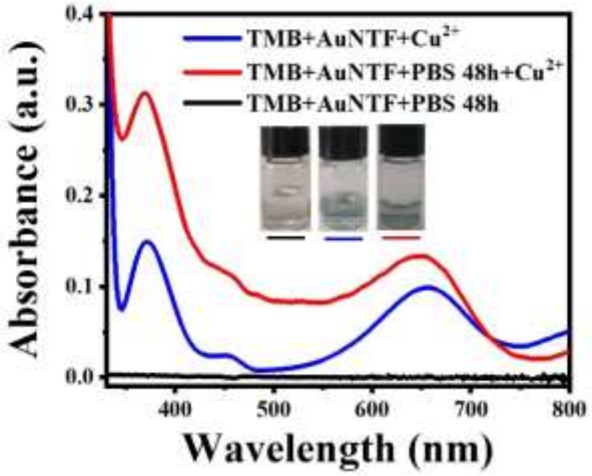

Figure S11. Copper dependent ROS gneration property of AuNTF nanoclusters before and after 48 h incubation in PBS determined by UV-Vis spectra and ROS indicator (TMB).

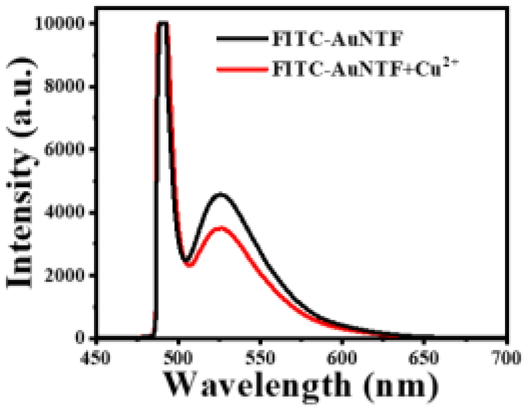

Figure S12. The fluorescence spectra of AuNTF nanoparticles with and without addition of the endogenous concentration of Cu^{2+} in 4T1 cells (1 mg/L).

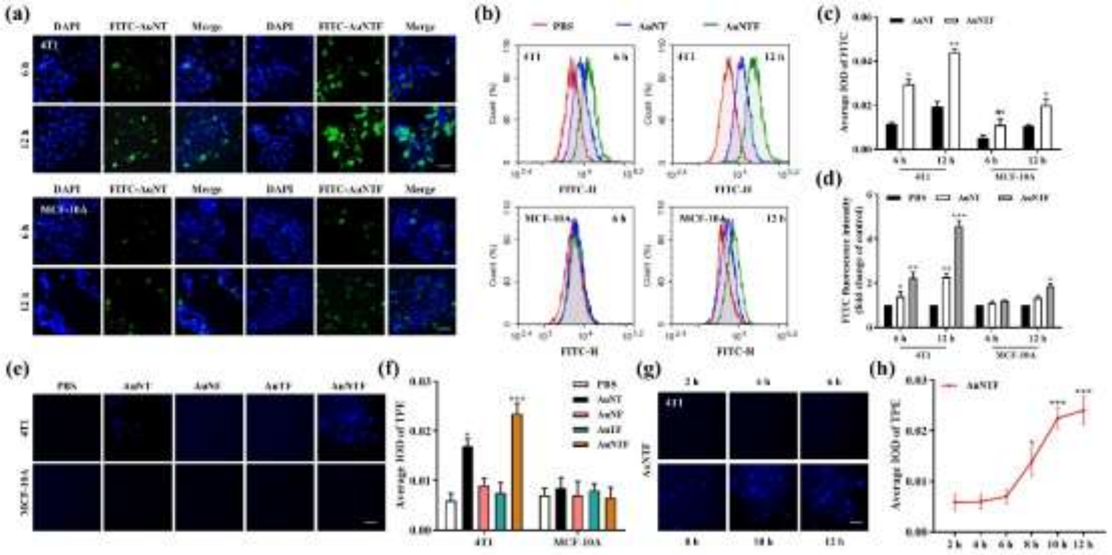

Figure S13. (a) Fluorescence microscopy images of the 4T1 and MCF-10A cells after incubation with FITC labeled AuNT and FITC labeled AuNTF nanoclusters for different time. The scale bar is 50 μm. **(b)** Flow cytometry analysis of the cellular uptake and retention of FITC labeled AuNT and FITC labeled AuNTF nanoclusters in 4T1 and MCF-10A cells. PBS served as a control group. **(c)** Quantitative analysis of Fig. S13a. **(d)** Quantitative analysis of Fig. S13b. **(e)** Fluorescence microscopy images of 4T1 and MCF-10A cells after incubation with PBS, AuNT, AuNF, AuTF and AuNTF for 12 hours. The scale bar is 50 μm. **(f)** Quantitative analysis of Fig. S13e. **(g)** Fluorescence microscopy images of 4T1 cells after incubation with AuNTF for different time. The scale bar is 50 μm. **(h)** Quantitative analysis of Fig. S13g. Data are presented as the

means ± SD (n = 3). The asterisks indicated that the differences between control groups are statistically significant using an ANOVA test (*$P < 0.05$, **$P < 0.01$, ***$P < 0.001$).

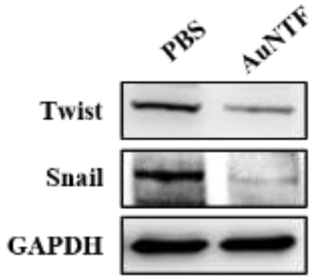

Figure S14. The expression of Twist and Snail in tumor cells after treatments of PBS and AuNTF evaluated by western blotting analysis.

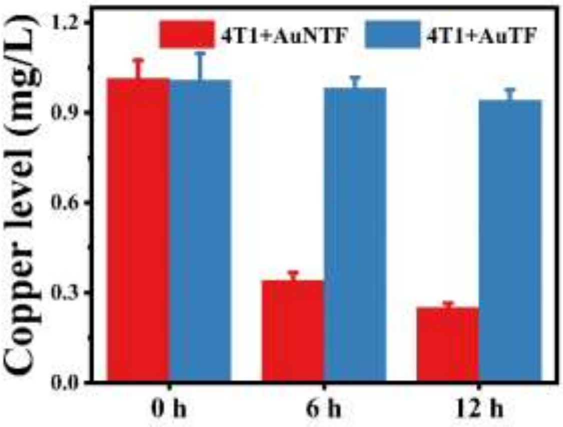

Figure S15. The concentration of copper ions in 4T1 cells during incubation with AuNTF or AuTF for 12 h.

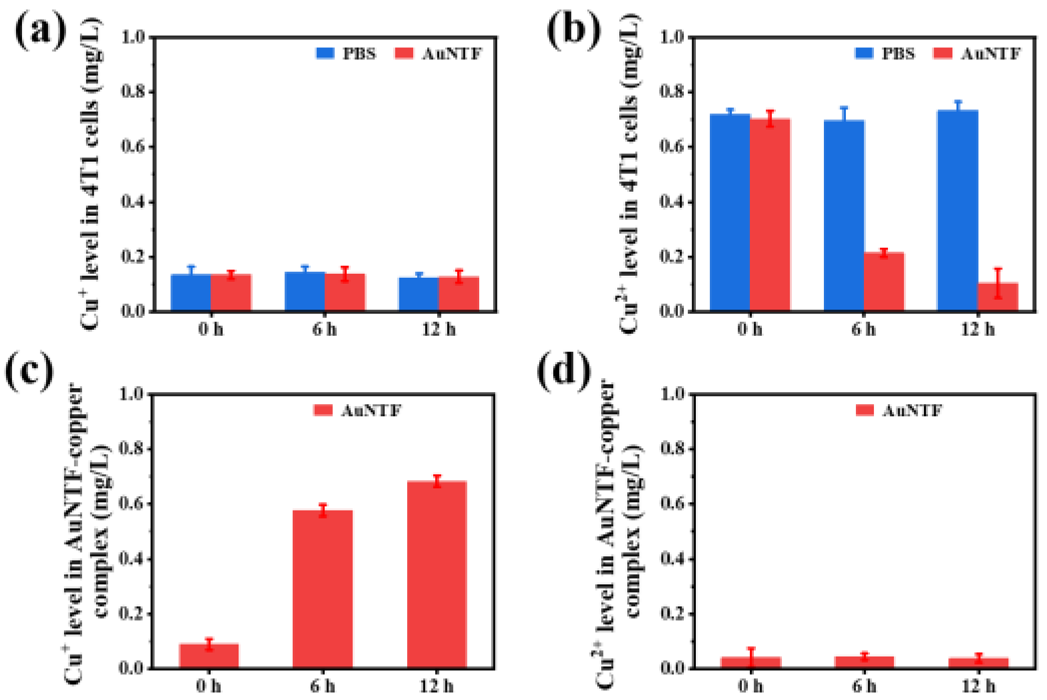

Figure S16. The intracellular Cu^{+} level **(a)** and Cu^{2+} level **(b)** in 4T1 cells within 12 h incubation with PBS or AuNTF, respectively. The Cu^{+} level **(c)** and Cu^{2+} level **(d)** in the AuNTF-copper complex, which were obtained by the cell lysate and centrifugation. The Cu^{+} level was detected by the typical Cu^{+} indicator (bathocuproine) and UV-Vis spectra. The Cu^{2+} level was calculated by the difference between the copper concentration and Cu^{+} concentration, in which the copper concentration was determined by atomic absorption spectrometry.

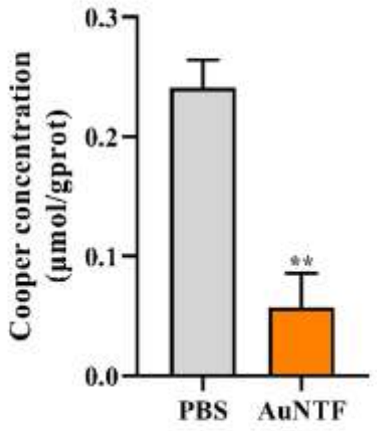

Figure S17. The Cu^{2+} level in 4T1 cells after 12 h incubation with PBS or

AuNTF, which measured by Cell Copper Colorimetric Assay Kit using the typical Cu^{2+} indicator DiBr-PAESA.

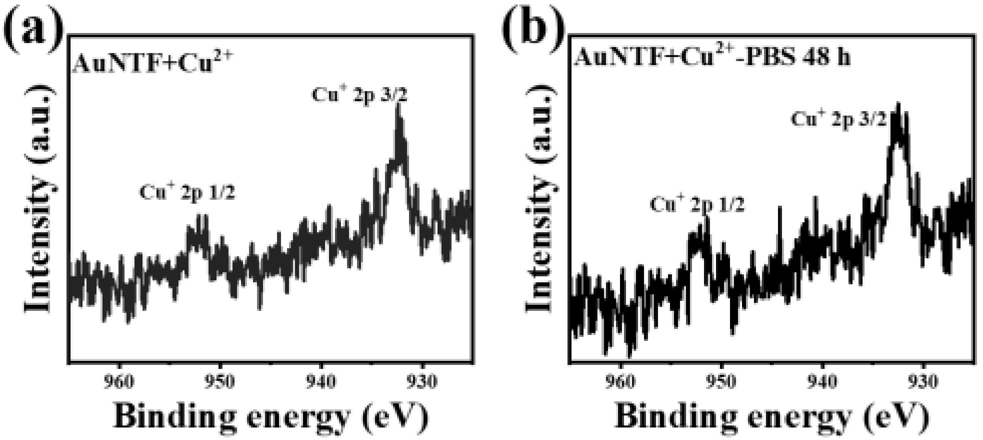

Figure S18. X-ray photoelectron spectroscopy (XPS) of Cu 2p obtained from $AuNTF+Cu^{2+}$ complex **(a)** before and **(b)** after incubation in PBS for 48 h.

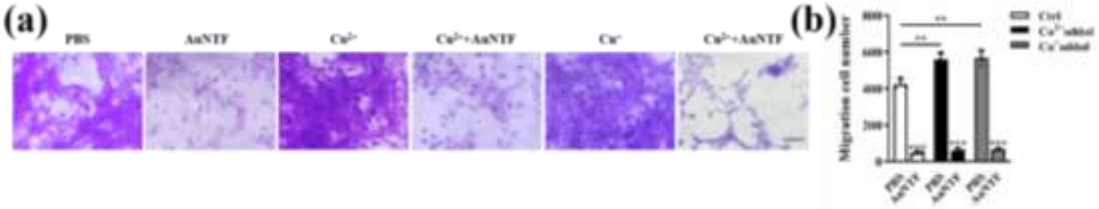

Figure S19. **(a)** Images and **(b)** quantitative analysis of 4T1 cells that migrated through the polycarbonate membrane, which were treated by various formulas and stained with 0.2% crystal violet. The scale bar is 100 μm.

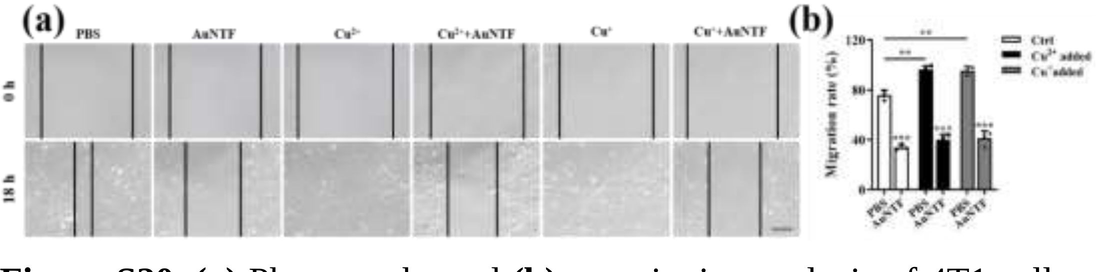

Figure S20. **(a)** Photographs and **(b)** quantitative analysis of 4T1 cells to indicate their migration in scratch assay after treatments by various formulas. The scale bar is 200 μm.

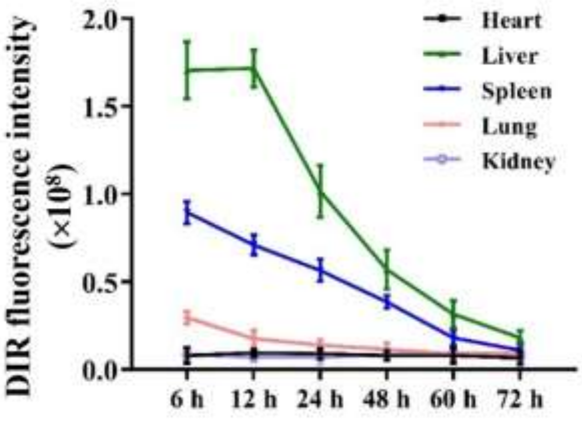

Figure S21. Quantification of DIR fluorescence intensity in heart, liver, spleen, lung and kidney at different time points after injection obtained from Fig. 6a. Data are presented as the means ± SD (n = 5).

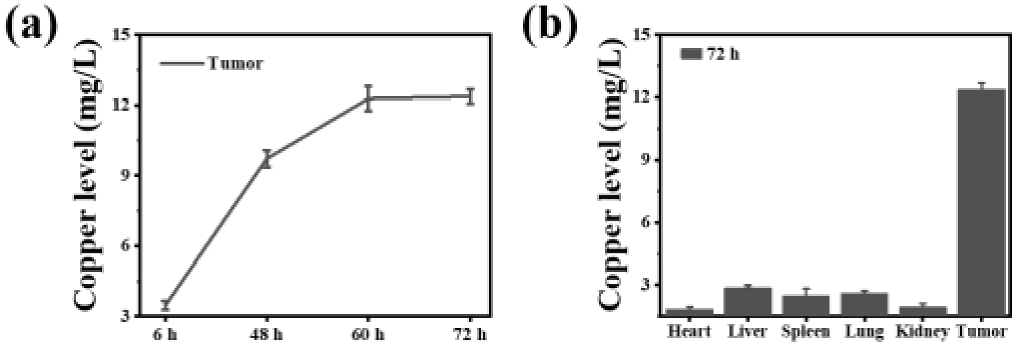

Figure S22. (a) The copper level in tumor after treatment by AuNTF at different time points. **(b)** The copper level in heart, liver, spleen, lung, kidney and tumor at 72 h after injection of AuNTF.

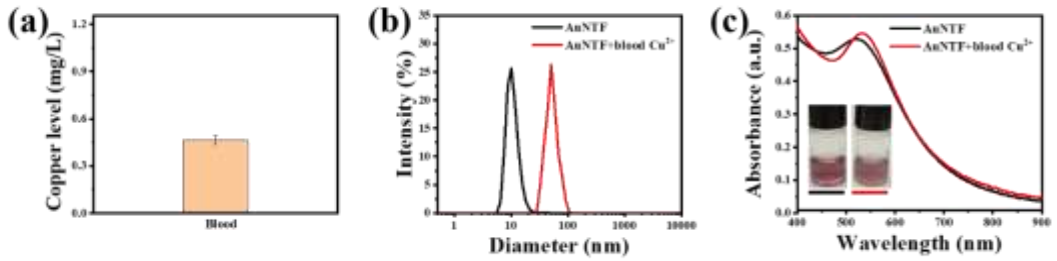

Figure S23. (a) The concentration of copper ions in blood. **(b)** The Dynamic Light Scattering (DLS) and **(c)** UV-Vis spectrum and photographs of AuNTF nanoparticles with and without addition of the endogenous concentration of Cu^{2+} in blood (0.48 mg/L).

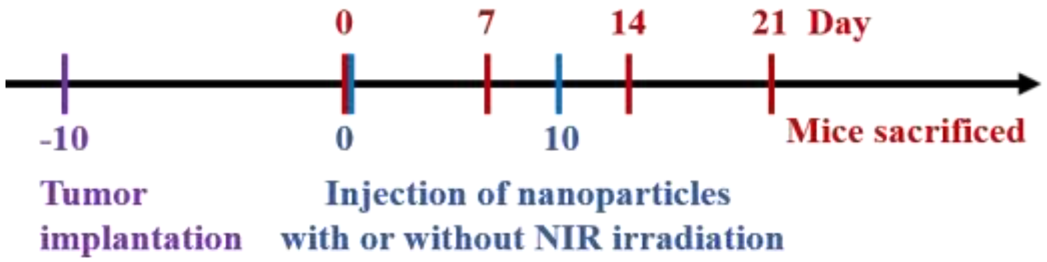

Figure S24. Schematic diagram of treatment regimen.

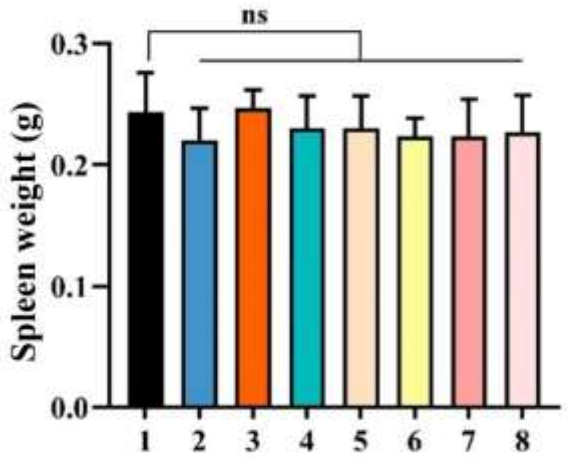

Figure S25. Weight of spleen harvested from tumor-bearing mice after different therapies at day 21. (1: PBS, 2: AuNT, 3: AuNT with NIR irradiation after injection of 60 h, 4: AuTF, 5: AuTF with NIR irradiation after injection of 60 h, 6: AuNTF, 7: AuNTF with NIR irradiation after injection of 60 h, 8: AuNTF with NIR irradiation after injection of 72 h.) Data are presented as the means ± SD (n = 5).

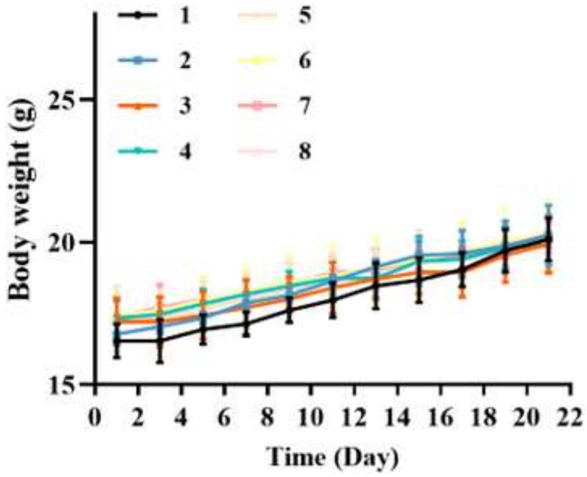

Figure S26. Time evolution of body weight of tumor-bearing mice after different therapies. (1: PBS, 2: AuNT, 3: AuNT with NIR irradiation after

injection of 60 h, 4: AuTF, 5: AuTF with NIR irradiation after injection of 60 h, 6: AuNTF, 7: AuNTF with NIR irradiation after injection of 60 h, 8: AuNTF with NIR irradiation after injection of 72 h.) Data are presented as the means ± SD (n = 5).

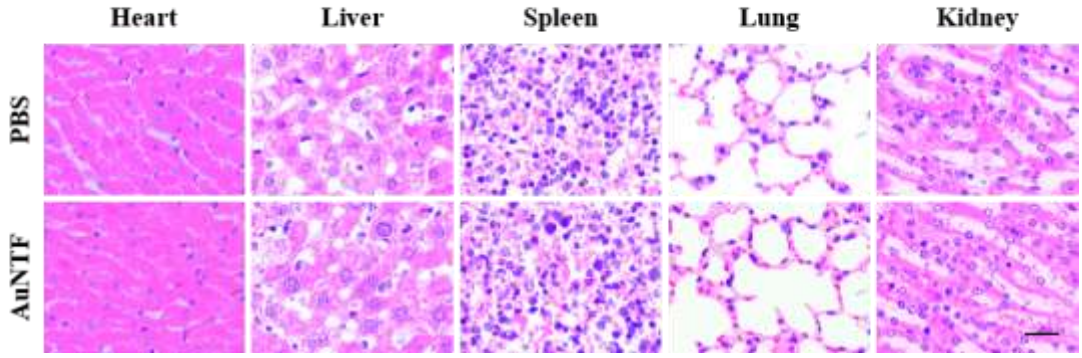

Figure S27. H&E staining of the heart, liver, spleen and kidney tissues obtained from tumor bearing mice after treatments by PBS and AuNTF.
